# Rational Engineering of Self-Supported Catalysts for High-Performance Electrochemical Oxygen Evolution and Ethylene Glycol Oxidation

**DOI:** 10.1021/acs.chemmater.6c00346

**Published:** 2026-05-13

**Authors:** Debabrata Bagchi, Gowtham Kenguva, Suptish Ghosh, Zishuo Zhang, Sagie Katz, Johannes Schmidt, Ingo Zebger, Tobias Sontheimer, Prashanth W. Menezes

**Affiliations:** † Department of Materials Chemistry for Catalysis, Helmholtz-Zentrum Berlin für Materialien und Energie GmbH, Hahn-Meitner-Platz 1, Berlin 14109, Germany; ‡ Department of Chemistry: Metalorganics and Inorganic Materials, 26524 Technische Universität Berlin, Straße des 17 Juni 135, Sekr. C2, 10623 Berlin, Germany; § Department of Chemistry: Biophysical Chemistry, Technische Universität Berlin, Straße des 17 Juni 115, Secr. PC14, 10623 Berlin, Germany; ∥ Functional Materials, Institute of Chemistry, Faculty II Mathematics and Natural Sciences, Technische Universität Berlin, 10623 Berlin, Germany; ⊥ Strategy Department of Energy and Information, Helmholtz-Zentrum Berlin für Materialien und Energie GmbH, Hahn-Meitner-Platz 1, Berlin 14109, Germany

## Abstract

Development of efficient electrocatalyst materials for performing both the oxygen evolution reaction (OER) and the electrochemical oxidation of ethylene glycol (EGOR) is crucial for advancing energy-efficient electrolysis and valorization of plastic waste-derived chemicals. In this study, we present a comprehensive investigation of self-supported Ni-based catalysts grown on nickel foam and systematically tuned with Mn, Fe, Co, and Pd to achieve controllable bifunctional activity. Among these materials, Fe-NiO_*x*_H_*y*_ exhibits superior OER performance in 1 M KOH, delivering an overpotential of 250 ± 7 mV at 100 mA cm^–2^ with a Tafel slope of 35 ± 2 mV/dec. Hierarchical architectures and their crystal structure, confirmed by scanning electron microscopy and X-ray diffraction, provide abundant active sites and enhance mass transport kinetics. Quasi in situ Raman spectroscopy and ex situ X-ray photoelectron spectroscopy reveal potential, reactant concentration and activation-dependent reconstruction of metal active sites, demonstrating how controlled tuning of metal oxidation states affects both OER activity and EGOR selectivity. Electrochemical activation enhances the valence of metal centers, enabling precise control over EGOR product selectivity. Pd incorporation stabilizes *C_2_ intermediates, favoring glycolate formation at low anodic potentials, while Mn-, Fe-, and Co-modified Ni promote a *C_1_ pathway leading to formate at relatively higher potentials, with Fe-NiO_*x*_H_*y*_ achieving a Faradaic efficiency (FE) of up to 86.2% toward formate. In contrast, Pd-NiO_*x*_H_*y*_/NF delivers a glycolate FE of up to 92.5%. Optimized reaction conditions, including applied potential, EG concentration, and activation protocol, allow selective production of either glycolate or formate during EGOR. This work provides an active site and mechanistic understanding connecting catalyst composition, activation, and oxidation-state dynamics to selectivity, providing a detailed insight for integrating PET-derived EG valorization with energy-efficient hydrogen production.

## Graphical abstract

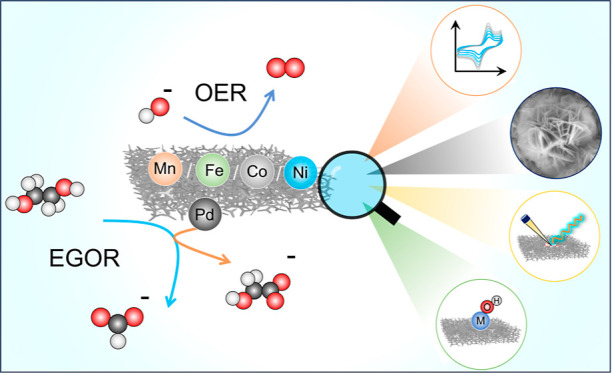

## 1 Introduction

The sustainable production of hydrogen (H_2_) from water electrolysis has become a central objective in the transition to low-carbon energy systems, as hydrogen offers high energy density, rapid kinetics at the cathode, and benign combustion products.^1–4^ Electrochemical water splitting consists of two half-reactions: the hydrogen evolution reaction (HER) at the cathode, generating H_2_, and the oxygen evolution reaction (OER) at the anode, producing O_2_.^5–9^ While HER occurs via a two-electron transfer process, carrying out OER efficiently remains challenging due to its sluggish kinetics and high overpotential requirements.^10,11^ Development of highly efficient, low-cost electrocatalysts that minimize OER overpotentials and sustain high current densities is therefore very crucial for advancing practical electrolyzers, particularly under alkaline conditions where earth-abundant materials can be utilized.^3,12–17^ At the same time, excessive use of plastics increases the accumulation of plastic waste in an alarming manner, with annual production going beyond hundreds of millions of tons and the vast majority being landfilled or released into the environment.^18–21^ This has led to environmental pollution, including the spread of microplastics in marine ecosystems and the release of toxic additives, causing long-term ecological and human health risks.^22,23^ Among various synthetic polymers, polyethylene terephthalate (PET) contributes a major fraction of plastic production. However, its recycling efficiency remains low and conventional mechanical recycling suffers from severe downcycling.^24,25^ Chemical recycling of PET via base-catalyzed or enzymatic depolymerization produces terephthalic acid and ethylene glycol (EG), which can be further upgraded electrochemically.^26,27^ Electrocatalytic EG oxidation reaction (EGOR) at the anode provides a promising alternative to conventional OER. By replacing water oxidation with EGOR, the overall cell energy input can be decreased, and at the same time, value-added organics such as glycolate and formate can be selectively produced while hydrogen is concurrently evolved at the cathode.^28–30^ This paired electrolysis approach has the potential to integrate waste upcycling with efficient H_2_ production via hybrid water electrolysis.^30^

Recently, there are few works that have reported on the electrochemical EGOR over transition-metal catalysts. However, most of the existing works have focused on catalytic activity rather than systematically investigating the factors that govern selectivity.^31–33^ For example, Ni- and Co-based materials have been shown to convert EG to formate with Faradaic efficiencies (FE) in the range of 60–85%,^33^ while certain Ag and Pd catalysts have yielded higher FE of glycolate under alkaline conditions.^34^ Similarly, Pt-alloy and Pt-cluster catalysts have exhibited higher selectivity toward glycolate production.^35^ Despite these advances, a detailed understanding of how key reaction parameters such as applied potential, catalyst activation, valence state of metal active sites, electrolyte composition, and local mass transport influence the competitive formation of formate versus glycolate remains limited.^36,37^ In this context, it is very important to understand how meticulous tuning of catalyst oxidation state and reaction conditions impacts product selectivity.^38^

Herein, we report a self-supported series of nickel-based catalysts (M-NiO_*x*_H_*y*_/NF, M = Mn, Fe, Co, Ni, Pd) grown directly on nickel foam (NF), acting as bifunctional electrodes for water oxidation and the upcycling of PET-derived ethylene glycol into value-added chemicals. Among these materials, Fe-NiO_*x*_H_*y*_/NF exhibits superior OER performance in 1 M KOH, delivering an overpotential of 250 ± 7 mV at 100 mA cm^–2^ with a Tafel slope of 35 ± 2 mV/dec. Incorporation of secondary metals into the NiO_*x*_H_*y*_ framework significantly tunes EGOR activity and selectivity by modulating the catalyst’s active sites under alkaline conditions. Notably, EGOR at higher potentials (e.g., 1.38 V vs RHE) promotes oxidation pathways leading to formate (FE > 90%). For formate production, Fe-NiO_*x*_H_*y*_, Mn-NiO_*x*_H_*y*_, and Co-NiO_*x*_H_*y*_ systems are more effective than Pd-NiO_*x*_H_*y*_, particularly after electrochemical activation, which increases the density of active metal oxyhydroxide species and significantly improves both FE and partial current density toward formate. On the other hand, Pd-containing electrodes favor glycolate formation with >90% selectivity at lower anodic potentials (e.g., 0.84 V vs RHE), where Pd remains in a reduced oxidation state stabilized by high ethylene glycol concentrations. By systematically correlating applied potential, electrolyte composition, catalyst activation, and metal identity and further mapping the evolution of surface species and dynamic active sites using quasi-in situ Raman spectroscopy, we establish well-defined reaction conditions for precise and reproducible control over EGOR selectivity between glycolate and formate.

## 2 Results and Discussion

### 2.1 Synthesis and Characterizations

A series of self-supported M-NiO_*x*_H_*y*_/NF catalysts (M = Mn, Fe, Co, Ni, and Pd) were synthesized via a versatile hydrothermal approach, enabling direct growth of binary metal hydroxide phases on NF; while Mn, Fe, Co, and Ni formed mixed hydroxide layers, Pd was reduced in situ to metallic species within the NiOH_*x*_ matrix due to its high standard reduction potential, facilitating uniform incorporation without classical hydroxide formation (Figure 1a).^39^ The powder XRD of the samples clearly showed that all the samples formation of layered double hydroxide structure (LDH) for Ni, Co, Mn, Fe, except Pd, which forms an additional metallic phase (Figure 1b). It is known that Pd has a more positive reduction potential, and it can take electrons from nickel and deposit in its elemental form. The scanning electron microscopy (SEM) image clearly showed that the morphology of LDH has been formed on the surface of NF, as for Mn, Fe, Co, and Ni cases (Figure 1c–f), with uniqueness in the morphology for each of the metals. It is found from that under similar hydrothermal conditions, Mn-NiO_*x*_H_*y*_/NF forms hierarchically assembled ultrathin nanosheet networks (Figure 1c), Fe-NiO_*x*_H_*y*_/NF forms into radially oriented nanoflower architectures (Figure 1d), Co-NiO_*x*_H_*y*_/NF develops urchin-like nanoneedle assemblies (Figure 1e), Ni-NiO_*x*_H_*y*_/NF exhibits compact nanoflake-based flower-like clusters (Figure 1f), and Pd-NiO_*x*_H_*y*_/NF have nanoparticle-aggregated morphologies (Figure 1g), reflecting metal-dependent hydrolysis kinetics, coordination environments, and lattice distortion effects that give rise to anisotropic nucleation and hierarchical growth on the NF substrate.^40–42^ SEM–EDX analysis and the corresponding elemental mapping (Figures S1–S5, Table S1) shows uniform distribution of all constituent elements, including the active metals, oxygen, and carbon. The absence of any kind of aggregation confirms the high homogeneity of the material, reflecting the meticulous control achieved during synthesis and provides an abundance of active sites across the sample.

**Figure 1:**
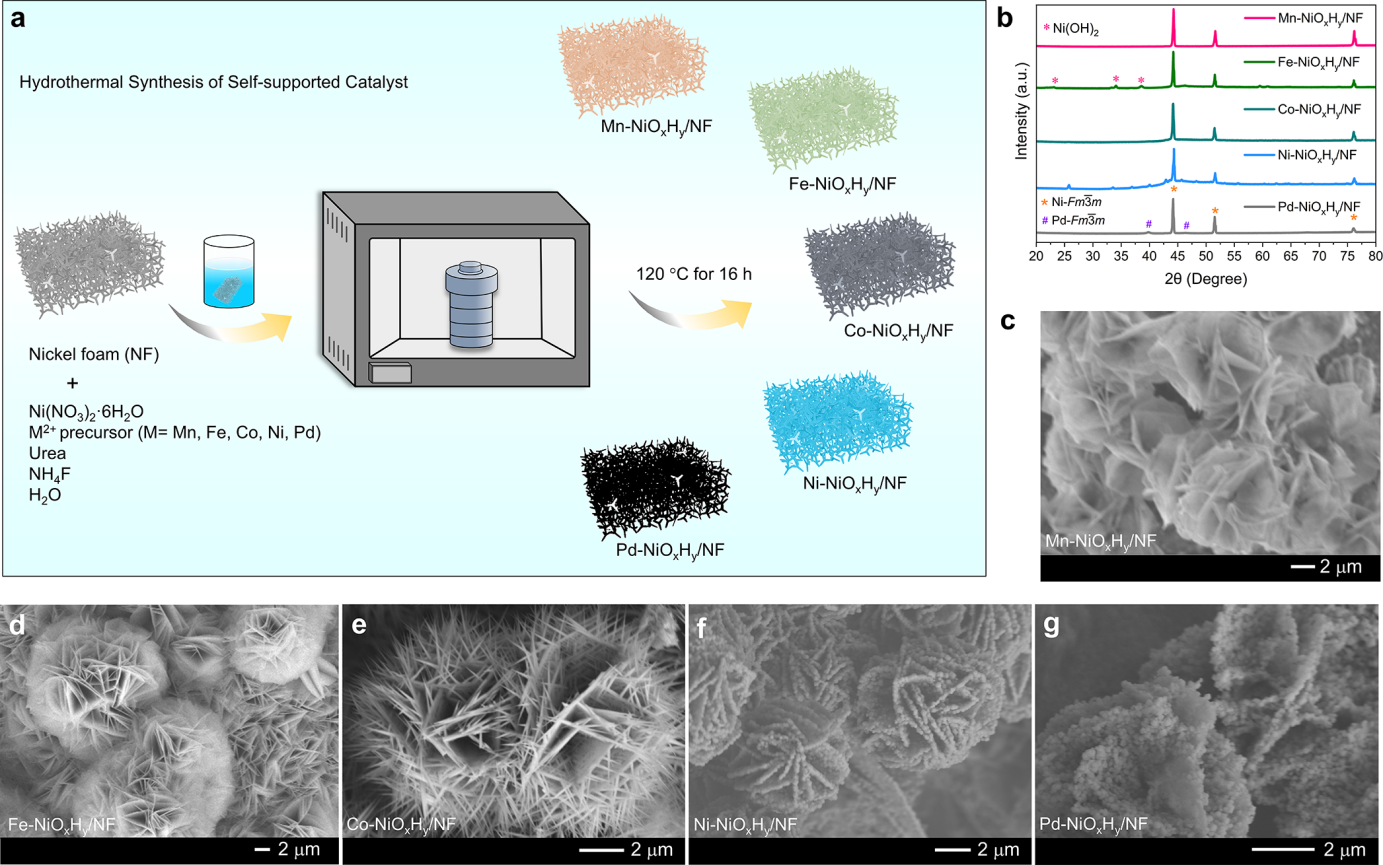
(a) Schematic illustration of the synthesis of the self-supported M-NiO_*x*_H_*y*_/NF (M = Mn, Fe, Co, Ni, Pd) catalyst via hydrothermal synthesis. (b) Powder XRD of the as-synthesized M-NiO_*x*_H_*y*_/NF self-supported catalyst grown on nickel foam (NF). SEM image of the as-synthesized materials grown on NF with variation of different metals like (c) Mn-NiO_*x*_H_*y*_/NF, (d) Fe-NiO_*x*_H_*y*_/NF, (e) Co-NiO_*x*_H_*y*_/NF, (f) Ni-NiO_*x*_H_*y*_/NF, and (g) Pd-NiO_*x*_H_*y*_/NF.

The incorporation of secondary metal species (M = Mn, Fe, Co, Ni, Pd) into Ni hydroxide architectures provides modifications in electronic structure and surface chemical compositions, as evidenced by Raman spectroscopy and X-ray photoelectron spectroscopy (XPS) (Figure 2). Raman spectroscopy suggests that the incorporation of such secondary metals (e.g., M = Mn, Fe, Co) induces changes in the local structure of the Ni hydroxide species (Figure 2a). While pure Ni^2+^ oxide or hydroxide species exhibit under the present condition only a relatively weak band around 432 cm^–1^, the incorporation of Mn, Fe, and Co leads to dominant vibrational features centered at approximately 455–460 cm^–1^ and 528–535 cm^–1^, corresponding to the Ni^II^–O bending and stretching modes in double layered or mixed hydroxides, respectively.^43–46^ The enhanced intensity of the Ni^2+^–O vibrational bands in the case of incorporation of Fe^3+^, Co^2+^, and Mn^2+^ can be attributed to heterometal-induced ordering of the Ni hydroxide lattice, where partial substitution of Ni by redox-active first–row transition metals promotes stronger metal–oxygen hybridization, increasing the local structural coherence, while enhancing also the polarizability of the constituent bonds through symmetry breaking and increased phonon–electron coupling.^47,48^ XPS survey spectra confirm the presence of Ni, O, and the respective secondary metals (Figure 2b). High-resolution Ni 2p spectra (Figure 2c) show the presence of two peaks, Ni 2p_3/2_ and Ni 2p_1/2_, located at ∼856 and 874 eV, respectively, which are characteristic features of Ni^2+^ with shakeup satellites corresponding to hydroxide species on the surface, which is consistent with the previous reports.^49,50^ The peaks at 643.4 and 652 eV can be attributed to Mn 2p_3/2_ and Mn 2p_1/2_ of Mn^3+^ (Figure 2d), respectively, confirming the presence of Mn in Mn-NiO_*x*_H_*y*_/NF with a valence state of 3+.^50^ The Fe 2*p*_3/2_ spectrum for Fe-NiO_*x*_H_*y*_/NF (Figure 2e) shows Fe^3+^ features at ∼711.0 eV, consistent with octahedrally coordinated Fe^3+^ incorporated into the Ni hydroxide lattice.^49^ The weak shoulder observed near 704–708 eV is attributed to multiplet splitting or background asymmetry.^51,52^ For Co-NiO_*x*_H_*y*_/NF, the Co 2p XPS consists of two main peaks, Co 2p_3/2_ and Co 2p_1/2_ at 780.9 and 796.6 eV, along with their satellite features, suggesting that cobalt is mainly present in the 2+ oxidation state (Figure 2f).^13,53^ The weak feature observed at ∼775 eV is assigned to the Co LMM Auger transition, whose presence and overlap with the Co 2p_3/2_ peak are characteristic of oxidized cobalt species (Co^2+^/Co^3+^) rather than metallic Co, providing additional confirmation of the nonmetallic chemical state of cobalt.^52^ For the Pd-NiO_*x*_H_*y*_/NF sample, Pd 3d spectra show a dominant Pd 3*d*_5/2_ signal at 333.8 eV, characteristic of metallic Pd^0,^ along with a weak feature at 336.25 eV corresponding to Pd^2+^ species (Figure 2g).^54^ This XPS feature indicates that Pd is predominantly present in its metallic state, with a minor fraction of partially oxidized Pd likely arising from interfacial interactions with neighboring Ni–O environments.^37^

**Figure 2:**
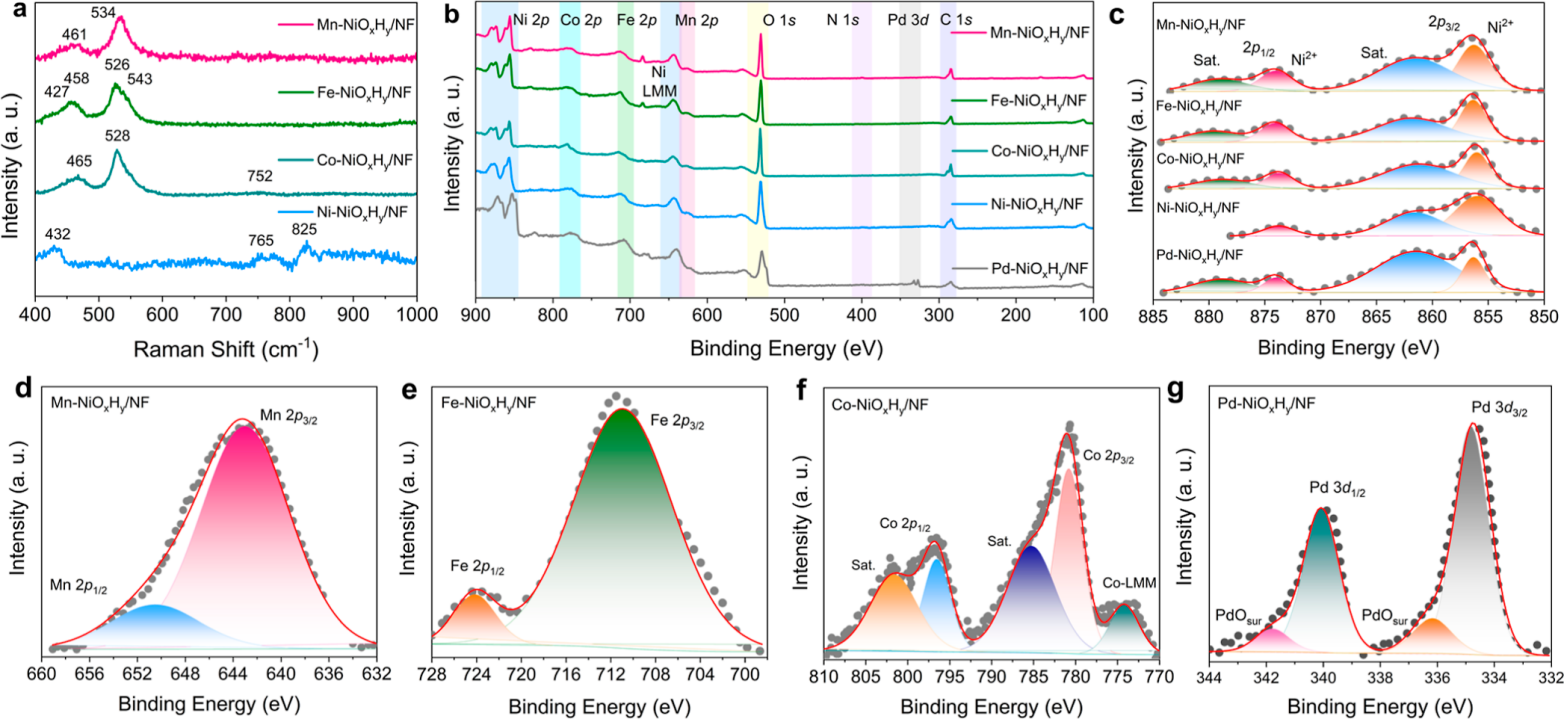
(a) Raman spectra of self-supported M-NiO_*x*_H_*y*_/NF electrodes (M = Mn, Fe, Co, Ni) acquired using a 407 nm excitation laser, highlighting vibrational features associated with metal–oxygen bonding and structural features. (b) XPS survey spectra of the as-prepared M-NiO_*x*_H_*y*_/NF electrodes, confirming the presence of the primary elemental constituents. High-resolution XPS spectra of (c) Ni 2p for all compositions, (d) Mn 2p for Mn-NiO_*x*_H_*y*_/NF, (e) Fe 2p for Fe-NiO_*x*_H_*y*_/NF, (f) Co 2p and Co-LMM for Co-NiO_*x*_H_*y*_/NF, and (g) Pd 3d for Pd-NiO_*x*_H_*y*_/NF, elucidating the oxidation states and electronic environments of the respective metal species.

Raman spectroscopy and XPS collectively demonstrate that the incorporation of secondary metal species into NiO_*x*_H_*y*_ matrices leads to systematic modification of the local coordination environment and electronic structure, evidenced by altered Ni–O vibrational responses and well-defined metal-specific oxidation states, confirming effective heterometal incorporation.

### 2.2 Electrocatalytic Oxygen Evolution Reaction (OER) and Ethylene Glycol Oxidation Reaction (EGOR) Performance

The initial electrochemical OER activities of the M-NiO_*x*_H_*y*_/NF (M = Mn, Fe, Co, Ni, Pd) electrodes were evaluated in 1.0 M KOH (Figure 3a). All electrodes show a well-defined Ni^2+^/Ni^3+^ redox transition at approximately 1.35–1.40 V vs RHE, followed by a sharp rise in anodic current attributed to OER onset (Figure S6a).^55,56^ Among these, Fe-NiO_*x*_H_*y*_/NF exhibits the best OER performance, requiring an overpotential of 250 ± 7 mV at a current density of 100 mA cm^–2^ (Figure 3b), outperforming Mn-NiO_*x*_H_*y*_/NF (370 ± 12 mV), Co-NiO_*x*_H_*y*_/NF (380 ± 12 mV), Pd-NiOH_*x*_/NF (400 ± 13 mV), and reference Ni-NiO_*x*_H_*y*_/NF (420 ± 14 mV) electrode. Overall, Fe-NiO_*x*_H_*y*_/NF demonstrates excellent OER activity, surpassing not only the other self-supported catalysts studied here but also most of the previously reported catalytic systems in similar condition (Figure S6b–d, Table S2).^57,58^ Electrochemical impedance spectroscopy (EIS) analysis reveals that Fe-NiO_*x*_H_*y*_/NF exhibits the lowest charge transfer resistance among the tested catalysts, indicating faster reaction kinetics (Figure S7).^59,60^ Fe-NiO_*x*_H_*y*_/NF also shows the smallest Tafel slope (35 ± 12 mV dec^–1^) than that of reference samples, indicative of accelerated OER kinetics (Figure 3c).^61,62^ It is important to mention that Pd-NiO_*x*_H_*y*_/NF exhibits a relatively larger Tafel slope (87 ± 4 mV dec^–1^), suggesting that despite higher conductivity due to metallic state of Pd, it does not stabilize OER intermediates and shows lower OER activity.^63,64^ This difference becomes very important during the investigation of EGOR. To characterize the electrode and to understand the morphological changes during the OER, the electrodes were subjected to cycling voltammetry (CV) cycling (1.10–1.52 V vs RHE, 20 mV s^–1^, 50 cycles) in 1.0 M KOH. Subsequently, the electrodes were characterized by SEM–EDX (Figures S8–S12) and powder XRD (Figure S13). For Mn-, Fe-, Co-, and Ni-based electrodes, the initial morphologies reconstruct into layered, sheet-like, and flower-type architectures characteristic of Ni­(M) oxyhydroxide phases formed under alkaline OER conditions.^65^ Elemental mapping confirms homogeneous distribution of the secondary metal within the reconstructed layers, and XRD shows the suppression of hydroxide reflections and the formation of broad features consistent with poorly crystalline NiOOH-type phases.^66,67^ In contrast, the Pd-NiO_*x*_H_*y*_/NF follows a different reconstruction pathway. The nanoparticle-based morphology is mostly retained after OER, along with partial formation of an oxygen-rich layer (Figure S12).^13^

**Figure 3:**
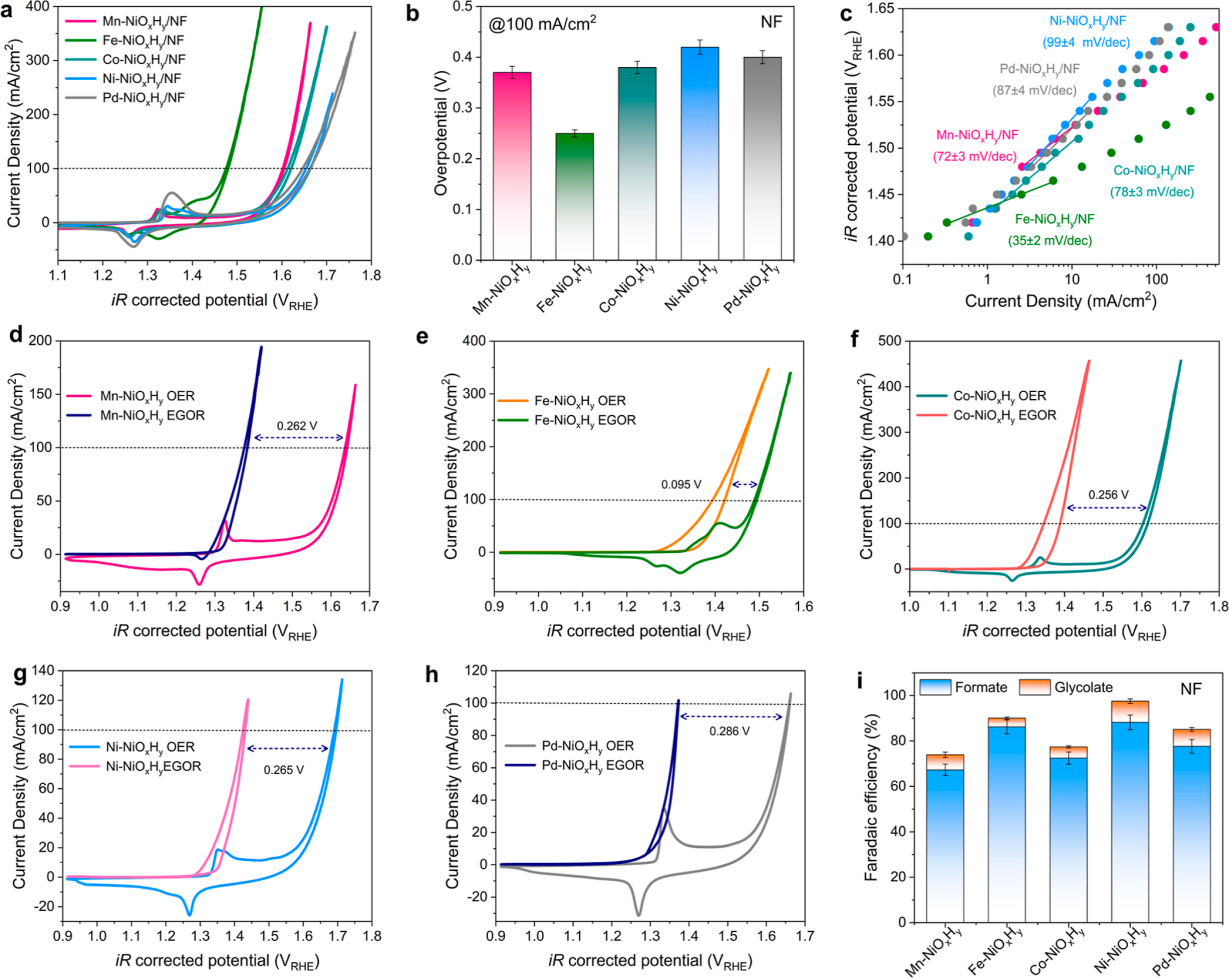
Electrocatalytic performance of self-supported M-NiO_*x*_H_*y*_/NF electrodes (M = Mn, Fe, Co, Ni, Pd) in alkaline media. (a) Comparison of cyclic voltammograms (CVs) recorded at a scan rate of 5 mV s^–1^ in 1.0 M KOH, highlighting the intrinsic OER activity of the catalysts. (b) Comparison of the overpotentials required to reach a current density of 100 mA cm^–2^, where Fe-NiO_*x*_H_*y*_/NF exhibits the lowest overpotential among the as-synthesized catalysts. (c) Tafel slopes derived from steady-state polarization measurements obtained by applying constant potentials for 300 s with a potential increment of 15 mV, providing insight into the OER kinetics. (d–h) CV responses of (d) Mn-NiO_*x*_H_*y*_/NF, (e) Fe-NiO_*x*_H_*y*_/NF, (f) Co-NiO_*x*_H_*y*_/NF, (g) Ni-NiO_*x*_H_*y*_/NF, and (h) Pd-NiO_*x*_H_*y*_/NF electrodes recorded in 1.0 M KOH in the absence and presence of 1.0 M ethylene glycol, illustrating the transition from the suppression of OER and the emergence of EGOR currents upon substrate introduction. All electrodes were initially activated under OER conditions prior to EGOR measurements via CV cycling in the potential window: 1.10–1.52 V vs RHE; scan rate: 20 mV s^–1^ for 50 cycles. (i) FEs of EGOR products (formate and glycolate) generated during bulk electrolysis conducted at 1.38 V vs RHE for 60 min of EG on the OER-activated M-NiO_*x*_H_*y*_/NF electrodes.

For the initial investigation of EGOR, the OER-activated catalyst was examined in the presence of 1.0 M EG dissolved in 1 M KOH, and a drastic enhancement of anodic current was observed for all electrodes (Figure 3d–h) as compared to the OER condition. In this case, the OER activation was performed to generate higher oxidation states of metals, which are widely recognized in the literature as being more reactive toward organic electrooxidation than their lower-valent counterparts.^28^ The high-valent metal centers formed under oxidative potentials have been found to facilitate small molecule oxidation by providing active metal sites with higher electron withdrawal and adsorption properties, a strategy that has improved performance in analogous electrooxidation of alcohol and biomass-derived compounds.^68–70^

During EGOR, the oxidation currents emerge at potentials as low as ∼1.30–1.35 V vs RHE, well below the OER onset. Quantitative product analysis (Figure 3i) with the help of ^1^H nuclear magnetic resonance (NMR, Figures S14 and S15) reveals that formate is the major product (FE of ∼80–90%) along with the formation of glycolate (FE of <5%) for all the activated catalysts when we perform EGOR at 1.38 V vs RHE for 60 min. To quantitatively assess activity and selectivity during EGOR, product formation rate (Figure S16a) and, most importantly, partial current densities (Figure S16b) were determined for all catalysts at 1.38 V vs RHE. Among the activated electrodes, Fe-NiO_*x*_H_*y*_/NF displays the highest formate partial current density (52 ± 2.5 mA cm^–2^), outperforming Mn-NiO_*x*_H_*y*_/NF (22 ± 2 mA cm^–2^), Co-NiO_*x*_H_*y*_/NF (23 ± 2 mA cm^–2^), and Pd-NiO_*x*_H_*y*_/NF (21 ± 2 mA cm^–2^), while the Ni-NiO_*x*_H_*y*_/NF reference shows the lowest activity (10 ± 2 mA cm^–2^). Notably, glycolate formation is negligible for all activated samples, indicating a strong preference for C–C bond-breaking oxidation pathways once the catalyst surface is electrochemically reconstructed. On the other hand, nonactivated catalysts exhibit significantly reduced formate selectivity and partial current densities (Figures S17 and S18), with Mn-NiO_*x*_H_*y*_ and Pd-NiO_*x*_H_*y*_ producing appreciable glycolate, suggesting stabilization of partially oxidized intermediates. Systematic variation of the OER activation method suggests that increasing the number of CV cycles prior to EGOR leads to an enhancement in partial current densities for both the major product formate (Figure S18d).

These results reveal that electrochemical activation is essential for high formate selectivity, while incorporation of secondary metals, particularly Fe, Mn, and Co, into the Ni matrix enhances conductivity and tunes the electronic structure of Ni oxyhydroxide phases, thereby enhancing the higher formate formation rates, which is comparable to previously reported studies (Table S3). Even Pd-containing catalysts, despite their intrinsic tendency toward partial oxidation, predominantly yield formate after activation, indicating the influence of reconstructed metal sites on the EGOR selectivity.^37,71^

### 2.3 Understanding the Origin of Glycolate Formation During EGOR

To understand the origin of glycolate formation and exclude the possibility of a chemical (or nonelectrochemical) transformation route, a series of control experiments were conducted under both chemical and electrochemical conditions (Figure 4a), and liquid products were analyzed by NMR. 1 M EG was first dissolved in 1 M KOH for 60 min in the absence of any catalyst, and we found no detectable amount of formate or glycolate. We have also performed the same treatments in the presence of as-prepared and OER-activated Pd-NiO_*x*_H_*y*_/NF electrodes without applying an external potential. In both catalyst-containing cases, only trace amounts of glycolate were detected, while no formate formation was observed, indicating that the chemical oxidation of EG to glycolate is minimal and does not proceed beyond partial oxidation under alkaline conditions. Notably, the extent of glycolate formation is suppressed when the activated catalyst is employed, suggesting that stabilization of partially oxidized intermediates has been reduced on the reconstructed surface. In contrast, electrochemical EGOR performed at 1.38 V vs RHE using the same catalysts leads to a pronounced increase in formate production exclusively for the activated electrode, confirming that formate generation is strictly electrochemically driven and highly dependent on OER-induced surface activation. Concentration-dependent studies reveal no pronounced dependence of formate selectivity on the EG concentration. However, lower EG concentrations (0.1 M) are marginally favored under high-potential EGOR conditions, forming a slightly enhanced formate selectivity (Figure 4b).

**Figure 4:**
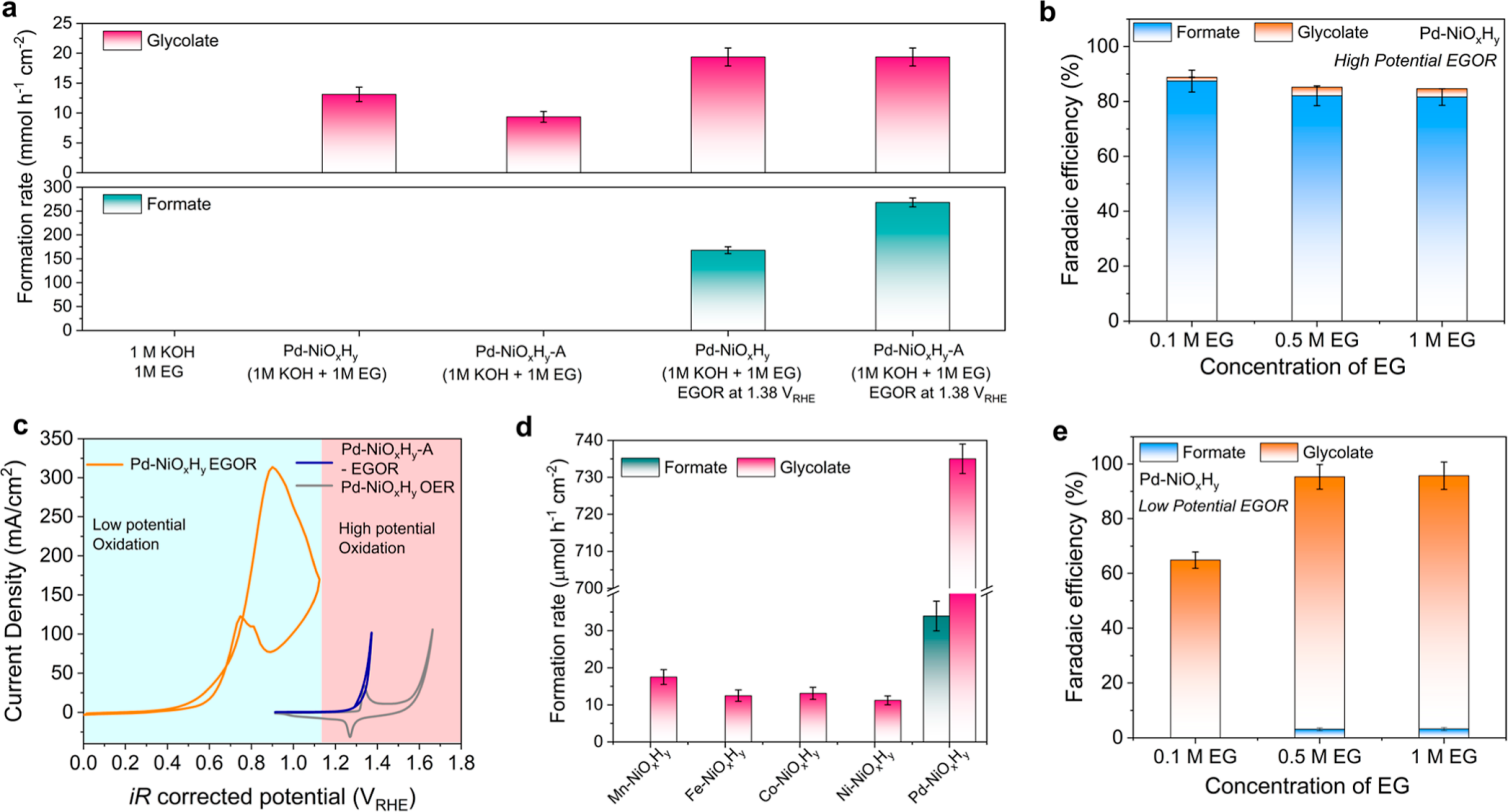
Mechanistic insights into EGOR and product selectivity on self-supported M-NiO_*x*_H_*y*_/NF electrodes. (a) Comparative analysis of formate and glycolate formation under chemical and electrochemical oxidation conditions, highlighting the influence of reaction pathways on product distribution. (b) Dependence of FE for formate and glycolate upon high potential (an applied potential of 1.38 V vs RHE, high potential), EGOR on EG concentration for the OER-activated Pd-NiO_*x*_H_*y*_/NF electrode. (c) Cyclic voltammograms of Pd-NiO_*x*_H_*y*_/NF recorded at low potentials (nonactivated catalyst) and high potentials with OER-activated catalyst, together with the corresponding OER CV, illustrating the impact of electrochemical activation on catalytic behavior. (d) Comparison of formation rates of formate and glycolate across different self-supported M-NiO_*x*_H_*y*_/NF catalysts (M = Mn, Fe, Co, Ni, Pd), emphasizing the role of metal identity in determining product selectivity. EGOR was performed in 1.0 M EG dissolved in 1.0 M KOH at an applied potential of 0.843 V vs RHE (low potential EGOR). (e) Dependence of FE for formate and glycolate on EG concentration for the as-prepared Pd-NiO_*x*_H_*y*_/NF electrode, highlighting the effect of substrate concentration on product selectivity during low potential EGOR.

After establishing the reaction protocol for formate formation at high potentials with the activated catalyst, we next investigated low-potential EGOR on as-prepared catalysts to find conditions favorable for partial oxidation pathways, especially for the formation of high-value product glycolate.^37^ Among all catalysts investigated here, Pd-NiO_*x*_H_*y*_/NF uniquely exhibits EGOR activity in the low-potential window of 0.4–1.2 V vs RHE (Figure 4c).

The oxidation peak observed for the Pd-NiO_*x*_H_*y*_/NF during the reverse scan is characteristic of Pd-mediated organic oxidation and originates from the oxidative removal of adsorbed carbonaceous intermediates accumulated during the forward scan, whereas it is absent in the activated catalyst due to the different reaction pathway and surface state governing ethylene glycol oxidation.^72^ Product analysis reveals that glycolate is formed exclusively (FE > 90%) on Pd-NiO_*x*_H_*y*_/NF under these conditions, whereas Ni-, Mn-, Co-, and Fe-based electrodes generate only trace amounts of glycolate with current densities below 1 mA cm^–2^, showing their activity is negligible by comparison (Figures 4d, and S19). Focusing on Pd-NiO_*x*_H_*y*_/NF, concentration-dependent studies conducted at 0.843 V vs RHE show that glycolate selectivity increases with increasing EG concentration, with a maximum FE of 92.5% in 1 M EG (Figure 4e), showing the distinct ability of Pd sites to promote selective low-potential, C–C bond-preserving oxidation of EG (Table S4). Collectively, we systematically investigated the key factors governing product selectivity toward glycolate and formate, including the choice of active metals, electrode activation, distinguishing chemical versus electrochemical contributions, applied potential, reactant concentration, and mass-transport effects (Figure 5).

**Figure 5:**
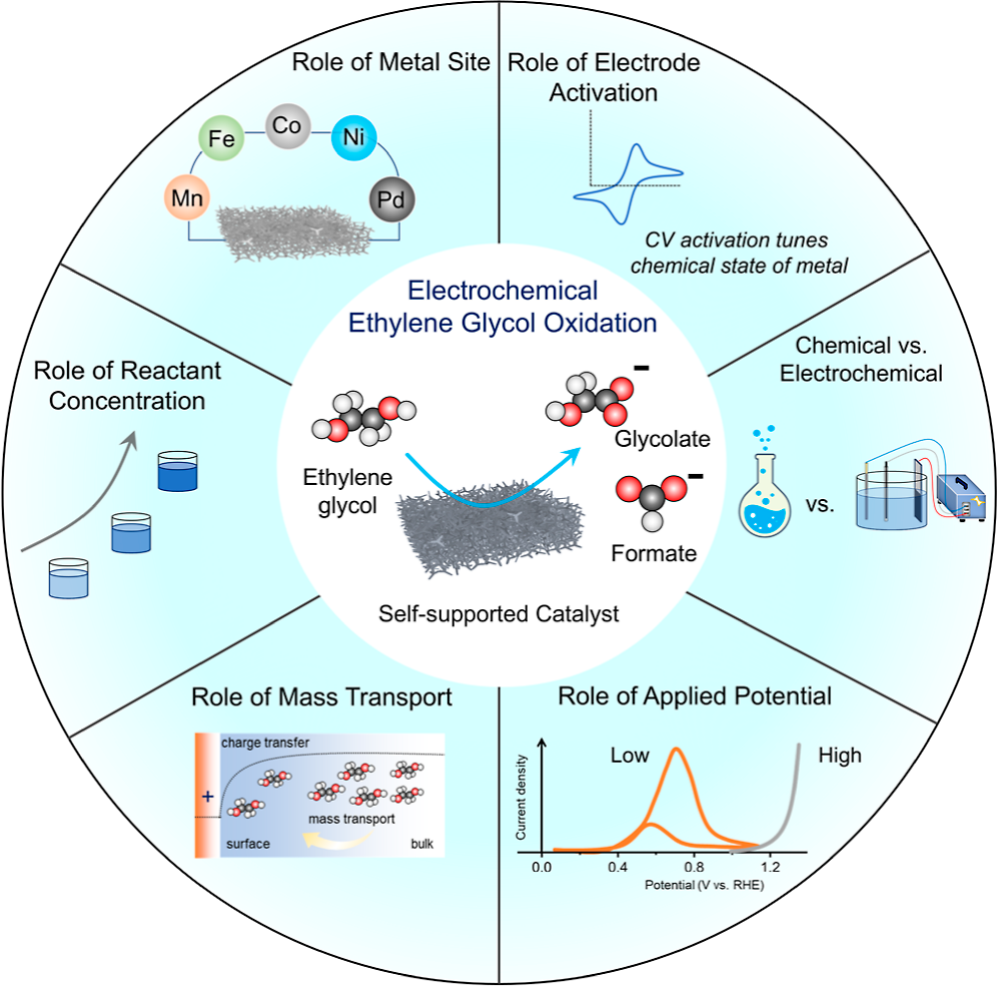
Effect of key reaction parameters on product selectivity and catalytic performance during EGOR studied in this work.

### 2.4 Probing the Active Site of the Catalyst at Different Reaction Conditions by Quasi In Situ Raman Spectroscopy

To understand the active site of the catalysts at different reaction conditions, we have performed a quasi-in situ Raman spectroscopic study by freeze-quenching samples under EGOR conditions in liquid nitrogen.^73^

The Raman spectra of OER-reconstructed electrodes (Figures 6a and S20) exhibit two dominating, strong Raman bands together with some overlapping signals of the related M^III^OOH species (M = Ni, Fe, Co) or oxides (Co^IV^, Mn^III^).^74,75^

**Figure 6:**
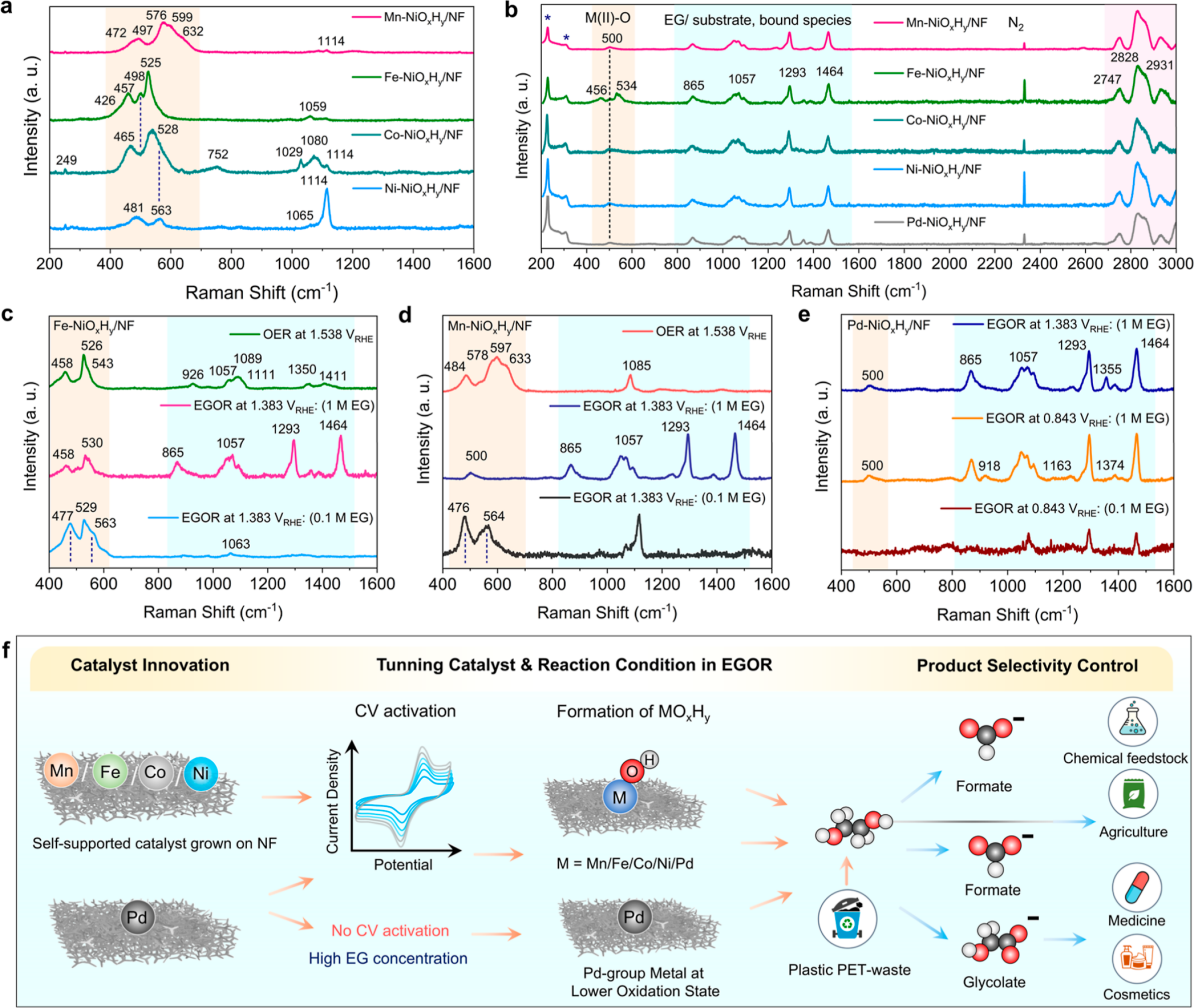
Quasi in situ and ex-situ Raman spectroscopic analysis of self-supported M-NiO_*x*_H_*y*_/NF catalysts (M = Mn, Fe, Co, Ni, Pd) to probe structural evolution and active sites during OER and EGOR. (a) Ex situ Raman spectra recorded using 407 nm wavelength of a Kr^+^ laser for excitation of OER-reconstructed electrodes for all M-NiO_*x*_H_*y*_/NF catalysts in 1.0 M KOH, illustrating metal–oxygen vibrational modes and structural modifications induced by electrochemical activation. (b) Quasi in situ Raman spectra recorded at 458 nm of M-NiO_*x*_H_*y*_/NF electrodes under EGOR conditions using preactivated OER electrodes, revealing changes in Ni–O bonding and surface species during EGOR. (Bands related to ice are marked with an asterisk.) (c) Comparative Raman spectra recorded at 458 nm of Fe-NiO_*x*_H_*y*_/NF under varying EG concentrations at high-potential EGOR (1.38 V vs RHE) and OER conditions, highlighting substrate-dependent structural responses. (d) Raman spectra recorded at 458 nm of Mn-NiO_*x*_H_*y*_/NF under high-potential EGOR (1.38 V vs RHE) across different EG concentrations and OER conditions, demonstrating metal-dependent modulation of active sites. (e) Raman spectra of Pd-NiO_*x*_H_*y*_/NF recorded at 458 nm under high-potential EGOR with OER-reconstructed catalyst (1.38 V vs RHE) and low-potential EGOR (0.843 V vs RHE) with as-prepared catalyst in varying EG concentrations. (f) Schematic illustration summarizing the tuning of product selectivity during EGOR through catalyst design, activation, and reaction condition optimization, identifying conditions that maximize formate and glycolate formation for targeted applications.

The band at ∼478–498 cm^–1^ corresponds to the depolarized E_g_ bending mode δ­(Co/Ni/Fe/^III^–O) and the band at 563–570 cm^–1^ is related to the polarized A_1g_ stretching mode ν­(Ni/Fe/Co^III^–O), indicating the formation of layered Fe/Co/Ni (oxy)­hydroxides.^33,44,76^ The broad peak between 800 and 1000 cm^–1^ along with peak at 1079 cm^–1^ for Fe-NiO_*x*_H_*y*_/NF seen at 458 nm in Figures 6b and S20 can be attributed to ν­(O–O) of the active oxygen species (NiOO^–^) in the NiOOH under OER.^44^ The peaks at around 1060–1070 cm^–1^ can be due to adsorbed CO_3_^2–^ coming from dissolved CO_2_ from ambient air in KOH electrolyte. For Co-NiO_*x*_H_*y*_/NF, two overlapping bands at ∼478 and ∼570 cm^–1^ could also be assigned to the E_g_ and A_1g_ vibrational modes of the high-valency Co­(IV) species (CoO_2_).^77^ For Mn-NiO_*x*_H_*y*_/NF, the Raman band centered at around 576 cm^–1^ corresponds to the in-plane Mn–O stretching vibration (A_1g_) along the octahedral layers and two bands at ∼500 and ∼632 cm^–1^ are associated with out-of-plane Mn–O vibrations (A_2g_) perpendicular to the layers of the birnessite phase of Mn^3+^, identified as the active phase. The band at 599 cm^–1^ belongs to the stretching vibrations of [Mn–O_6_] units.^78^

To map the evolution of vibrational features of different bonds related to catalyst active sites and adsorbed species and possible intermediates during EGOR, Raman spectra of standard references (ethylene glycol, glycolate, formate as substrate and potential products, respectively) and the bare NF substrate under EGOR conditions and just with EG were recorded (Figure S21). In the quasi in situ Raman spectra on Ni-based electrodes during EGOR, the appearance of distinct bands at ∼865, ∼1057, ∼1293, and ∼1464 cm^–1^ was observed (Figure 6b). When adding EG on bare NiF without any electric connection (Figure S21), only minor variations in the latter two signals are found. This suggests that all four bands are mainly related to surface-bound EG species, as observed prior on Si and Ni substrates, yielding very similar spectra compared to dissolved EG in aqueous solutions.^79^ Slight variations from our own reference spectra in solution, Figure S21, might be related to conformational changes or alteration of the resonance enhancement of the Raman scattering of the surface-bound EG. Although spectral contribution/overlaps with surface-adsorbed organic fragments evolving during the oxidation reaction cannot be excluded, they are expected to be rather weak due to their limited stability. The 865 cm^–1^ feature likely corresponds to C–C stretching and C–C–O bending in adsorbed ethylene glycol. The bands around ∼1057 cm^–1^ correspond to C–O stretching modes. The bands at ∼1293 and ∼1464 cm^–1^ can be assigned to CH_2_ deformation/scissoring and twisting modes.^79–81^ As shown before in the combined electrochemical and NMR study, the reaction progresses, and C–C cleavage becomes favorable in the higher potential case. These surface C_2_ species break down into C_1_ fragments, with formate (HCOO^–^) emerging as the dominant product in alkaline media. In alkaline EGOR, both C_2_ intermediates (e.g., glycolate, glyoxal-type species) and C_1_ species such as formate and carbonate have been reported by spectroscopic studies, pointing to parallel oxidation pathways with formate favored under basic conditions.^82^

For Fe-NiO_*x*_H_*y*_/NF, in situ Raman features at 477 and 563 cm^–1^, bending [δ­(Ni^III^–O)] and stretching [ν­(Ni^III^–O)] characteristics, respectively, of high-valent Ni–Fe oxyhydroxide species (γ-NiOO_*x*_H_*y*_) are more pronounced at lower EG concentration (0.1 M). This indicates that higher EG concentrations promote the reduction of Ni^3+^ species in the oxyhydroxide phase to Ni^2+^, as reflected by the Raman bands at ∼457 and ∼529 cm^–1^ of the LDH layer with Fe (Figure 6c).^83^ Mn–Ni_*x*_OH_*y*_/NF exhibits similar in situ Raman features at ∼476 cm^–1^ (Ni–O/Mn–O bending, E_g_) and ∼ 564 cm^–1^ (Ni–O/Mn–O stretching, A_1g_) at low EG concentration, indicating the formation of high-valent oxyhydroxide species under the same EGOR conditions (Figure 6d). An additional Raman band at ∼918, observed on Pd-NiO_*x*_H_*y*_/NF at low potential, might be related to some intermediate species, which cannot be assigned unambiguously at the moment.^84^ The selective formation of glycolate on Pd-NiO_*x*_H_*y*_ at lower anodic potentials can be rationalized by electronic structure considerations.

Incorporation of Pd is expected to downshift the effective d-band center of the surface ensemble, thereby weakening the adsorption strength of strongly oxidized carbonaceous intermediates and suppressing C–C bond cleavage. In contrast, Fe/Co/Mn incorporation stabilizes high-valent Ni^3+^/Ni^4+^ oxyhydroxide species with stronger Ni–O covalency, promoting deeper oxidation and favoring the C_1_ pathway toward formate at higher potentials, consistent with our quasi in situ Raman spectroscopic observations.^85–87^

## 3 Conclusion

This work demonstrates a rational approach to engineering self-supported Ni-based electrodes that enables controlled modulation of anodic reaction pathways in alkaline media. The systematic incorporation of secondary metal species into the NiO_*x*_H_*y*_ matrix induces distinct electronic and structural effects that govern both OER and EGOR behavior. While Fe/Co/Ni/Mn incorporation enhances the OER kinetics, Fe is the most effective by stabilizing high-valent Ni oxyhydroxide and favoring formate formation via EGOR. Pd-modified NiO_*x*_H_*y*_ selectively promotes EGOR at lower anodic potentials with a high FE toward glycolate, providing a clear decoupling of OER activity and EGOR selectivity (Figure 6f). Quasi-in situ Raman analysis reveals that electrochemical activation and dynamic surface reconstruction are central to defining the catalytically active state, with metal-dependent modulation of Ni–O motifs dictating substrate adsorption, while combined NMR with electrochemical studies revealed insights into the reaction selectivity from C_2_ to C_1_ products. Optimal glycolate production on Pd requires Pd in a low-valent state and a high local concentration of EG at the electrode–electrolyte interface. The ability to selectively suppress the OER while driving EGOR at higher current densities provides a viable strategy for lowering anodic energy input while enabling coupled chemical upgrading. Notably, these findings establish detailed insights into designing multifunctional anodic catalysts, providing generalizable principles for integrating hydrogen production with selective organic electrosynthesis.

## 4 Experimental Section

### 4.1 Chemicals and Reagents

The following chemicals were used for the synthesis without further purification: nickel­(II) nitrate hexahydrate (Ni­(NO_3_)_2_·6H_2_O, 99%, Acros), ammonium fluoride (NH_4_F, 98%, Sigma-Aldrich), urea (CH_4_N_2_O, 99%, Alfa Aesar), palladium chloride (PdCl_2_, 99%, Sigma-Aldrich), manganese­(II) chloride tetrahydrate (MnCl_2_·4H_2_O, 98%, TCI America), nickel­(II) chloride hexahydrate (NiCl_2_·6H_2_O, 98%, Sigma-Aldrich), cobalt­(II) chloride hexahydrate (CoCl_2_·6H_2_O, 98%, Acros), and iron­(II) chloride tetrahydrate (FeCl_2_·4H_2_O, 98%, Sigma-Aldrich). Potassium hydroxide (KOH, 85%, Sigma-Aldrich) was used as the electrolyte, and all solutions were prepared using distilled water. Millipore water of conductivity 18.2 MΩcm was used for the synthesis and all other studies. The electrode substrate, nickel foam (NF), and fluorine-doped tin oxide (FTO, resistivity 8–12 Ω/sq) were purchased from Recemat BV and Sigma-Aldrich, respectively.

### 4.2 Hydrothermal Synthesis of Self-Supported M-NiO_*x*_H_*y*_/NF (M = Mn, Fe, Co, Ni, and Pd) Catalyst

The self-supported M-NiO_*x*_H_*y*_/NF electrodes were fabricated via a facile hydrothermal approach, ensuring intimate integration of the active metal-based catalyst on nickel foam for enhanced electronic conductivity and structural robustness. The synthesis was initially optimized for the Fe-NiO_*x*_H_*y*_/NF system. Briefly, nickel nitrate (Ni­(NO_3_)_2_·6H_2_O, 2.0 mmol), iron nitrate (FeCl_2_·4H_2_O, 0.5 mmol), ammonium fluoride (NH_4_F, 10.0 mmol), and urea (NH_2_CONH_2_, 25.0 mmol) were dissolved in 40 mL deionized water to form a homogeneous precursor solution. The solution chemistry was carefully tuned to favor controlled hydrolysis and coprecipitation of Ni^2+^ and Fe^2+^ species under hydrothermal conditions, with urea serving as a slow-release hydroxide source via thermal decomposition and NH_4_F acting as a morphology-directing agent by moderating nucleation and growth. Prior to hydrothermal treatment, nickel foam (10 × 40 mm) was subjected to sequential sonication in acetone, 3 M HCl, and ethanol to remove surface contaminants and native oxides, followed by thorough rinsing with deionized water. The pretreated foam was immersed in the precursor solution within a 45 mL Teflon-lined stainless-steel autoclave, which was then sealed and heated at 120 °C for 16 h. During this hydrothermal process, in situ generation of OH^–^ ions from urea decomposition facilitated the formation of mixed Ni–Fe hydroxide nanosheets directly on the nickel substrate. Following the reaction, the autoclave was allowed to cool naturally to room temperature, and the modified foam was recovered, extensively washed with deionized water and ethanol, and dried at 60 °C for 2 h. This strategy produced a uniform, mechanically robust Fe-NiO_*x*_H_*y*_ layer with high surface area, ensuring efficient electrolyte accessibility and enhanced catalytic performance. Similar procedures were employed for the synthesis of other binary M-NiO_*x*_H_*y*_/NF systems (M = Mn, Co, Ni, Pd). In each case, the corresponding metal precursor (MnCl_2_·4H_2_O, CoCl_2_·6H_2_O, NiCl_2_·6H_2_O, or PdCl_2_) replaced Fe­(NO_3_)_3_ in equimolar ratios while maintaining identical urea and NH_4_F concentrations, reaction volume, and hydrothermal conditions to ensure comparability across the series. For Pd-NiO_*x*_H_*y*_/NF, it is noted that classical layered double hydroxide formation is not favored due to the different coordination chemistry of Pd^2+^ and its reduction potential; nevertheless, a homogeneous deposition of Pd species within the NiO_*x*_H_*y*_ matrix was achieved, producing a highly conductive, self-supported catalytic layer.

The washing protocol postsynthesis was carefully performed to remove unreacted salts and loosely bound particulates without disrupting the nanosheet morphology. The stepwise immersion in multiple deionized water and ethanol was carried out. The final drying at moderate temperature (60 °C) prevented structural collapse, producing mechanically stable M-NiO_*x*_H_*y*_/NF electrodes suitable for subsequent electrochemical characterization. This hydrothermal strategy offers a general and versatile route to fabricate a series of binary M-NiO_*x*_H_*y*_/NF electrocatalysts with controlled composition, uniform coverage, and tunable morphology. The mass of the deposited M-NiO_*x*_H_*y*_ catalyst was determined by measuring the nickel foam before and after hydrothermal synthesis (the approximate mass loadings were, for Mn-NiO_*x*_H_*y*_/NF – 1.6 ± 0.05 mg/cm^2^; for Co-NiO_*x*_H_*y*_/NF – 1.3 ± 0.1 mg/cm^2^; Fe-NiO_*x*_H_*y*_/NF – 2.0 ± 0.1 mg/cm^2^; Ni-NiO_*x*_H_*y*_/NF – 1.6 ± 0.1 mg/cm^2^; Pd-NiO_*x*_H_*y*_/NF – 2.1 ± 0.05 mg/cm^2^).

### 4.3 Synthesis of NiOOH

NiOOH was synthesized by dispersing Ni­(OH)_2_ in 30 mL of a 4 M KOH solution, which was gradually heated to 45 °C. An excess amount of K_2_S_2_O_8_ was then added to the solution while maintaining the temperature at 45 °C for 18 h, during which the material transitioned to a black color. The black precipitate was washed thoroughly three times with 50 mL of deionized water, followed by a single rinse with 50 mL of acetone. The product was dried at 120 °C overnight under ambient conditions.^88^

### 4.4 Synthesis of FeOOH

FeOOH was synthesized by dissolving 500 mg (1.80 mmol) of Fe­(NO_3_)_3_·*x*H_2_O in 10 mL of deionized water. To this solution, 4 mL of 1 M NaOH (4 mmol) was added dropwise while stirring at room temperature for 30 min, resulting in a dark brown suspension. The mixture was then gently heated to 45 °C, and 2 mL of 30% H_2_O_2_ was added dropwise. Stirring was continued at 45 °C for an additional 18 h. The resulting orange suspension was centrifuged, and the precipitate was washed three times with deionized water before drying at 60 °C overnight in air.^89,90^

### 4.5 Synthesis of Co­(OH)_2_

Co­(OH)_2_ was prepared by adding 50 mL of 0.1 M NaOH solution dropwise to 80 mL of 0.05 M Co­(NO_3_)_2_ solution while stirring continuously at 45 °C for 2 h. This process yielded a pink precipitate, which was collected by centrifugation, washed three times with 50 mL of deionized water, and dried at 60 °C in air.^88^

### 4.6 Synthesis of NiFeO_*x*_H_*y*_

A mixed hydroxide, NiFeO_*x*_H_*y*_, was synthesized by dissolving 1 mmol each of Ni­(NO_3_)_2_·6H_2_O and Fe­(NO_3_)_3_·*x*H_2_O in 10 mL of deionized water. To this solution, 4 M KOH was added dropwise with vigorous stirring until precipitation was complete. The precipitate was aged at room temperature for 2 h, separated by centrifugation, and washed thoroughly with deionized water. This washing process was repeated three times to ensure purity. The washed precipitate was dispersed in 15 mL of water, followed by the immediate addition of 2 mL of 30% H_2_O_2_. The suspension was allowed to age overnight, yielding a dark red precipitate. The product was subsequently washed and dried at 60 °C in an oven.^88^

### 4.7 Characterization

#### 4.7.1 Powder X-ray Diffraction (PXRD)

Powder X-ray diffraction (PXRD) patterns were measured on a Bruker AXS D8 advanced automatic diffractometer equipped with a position-sensitive detector (PSD) and curved germanium (111) primary monochromator using Cu–K_α1_ radiation (λ = 1.5418 Å). The structural models of as-prepared products were constructed using the DIAMOND program version 3.0. The PXRD of the FTO deposited electrodes was measured using Bragg–Brentano geometry under air, using a Rigaku SmartLab 3 kW diffractometer (Rigaku Corporation, Japan) with Cu–K_α1_ (λ = 1.5418 Å) radiation. Data acquisition was carried out using the SmartLab Guidance software package Rigaku Corporation, Japan; Version 2.1.0.0. The sample of high entropy alloy was characterized with PXRD on a STOE Stadi-P with Cu-K_α1_ source (λ = 1.54058 Å), curved Ge(111) monochromator, and DECTRIS MYTHEN 1K detector. Phase analysis was performed using the structure model from the ICSD database.

#### 4.7.2 Scanning Electron Microscopy (SEM) and Energy Dispersive Spectroscopy (EDS)

Microstructural characterization was performed in a Zeiss Gemini 500 microscope integrated with Energy-dispersive X-ray spectroscopy (EDS) (EDAX, Apollo XPP). For mapping and elemental analysis, the SEM measurements were performed using a 15 kV electron gun energy integrated with an energy dispersive X-ray detector (Bruker Quantax XFlash 6|60). Data handling and analyses were attained with the software package EDAX.

#### 4.7.3 X-ray Photoelectron Spectroscopy (XPS)

X-ray photoelectron spectroscopy (XPS) measurements were carried out on a Kratos Axis Ultra X-ray photoelectron spectrometer (Kratos Analytical Ltd., Manchester, U.K.) using an Al–Kα monochromatic radiation source (1486.7 eV) with a 90° takeoff angle (normal to analyzer). The vacuum pressure in the analysis chamber was kept at 2 × 10^–9^ Torr. The XPS spectra were collected for C 1s, O 1s, Ni 2p, Co 2p, Mn 2p, Fe 2p, and Pd 3d levels with a pass energy of 20 eV and a step of 0.1 eV. The binding energies were calibrated relative to the C 1s peak energy position as 284.8 eV. Data analyses were conducted using Casa XPS (Casa Software Ltd.) and the vision data processing program (Kratos Analytical Ltd.).

#### 4.7.4 Raman Spectroscopy

The resonance Raman (RR) spectroscopy measurements were conducted using both the 458 and 407 nm excitation wavelengths of an Ar^+^ and a Kr^+^ gas Coherent laser. For spectra acquisition, the laser power was set to 1 mW. The temperature of the sample was kept at 80 K using a liquid N_2_ cooled cryostat from Linkam Scientific Instruments. A LabRam HR-800 Jobin Yvon confocal Raman spectrometer connected to a liquid-N_2_-cooled CCD detector was used to record the respective spectra. The experimental RR spectra are an average of 10–15 individual spectra, each obtained after an accumulation time of 60–120 s. The frequency calibration for the Raman spectra was done using Toluene as an external standard.

### 4.8 Electrochemical Measurements

#### 4.8.1 General Electrochemical Setup

The electrochemical measurement was carried out in the Biologic (SP 300) electrochemical workstation controlled by the EC lab software (version 11.50 software package). The activity of the catalyst was measured in a typical three-electrode cell setup. Platinum wire (0.5 mm diameter and 230 mm length), Hg/HgO (CH Instruments, Inc.; in 1 M NaOH), and the synthesized catalysts were used as a counter electrode, reference electrode, and working electrode, respectively. Commercially purchased 1 M aqueous KOH was used as the electrolyte (99.99% pure, Fe containing <0.05 ppm from ICP-AES). The electrochemical process was performed by cyclic voltammetry (CV), linear sweep voltammetry (LSV), and chronopotentiometry (CP) techniques in a 1 M KOH solution at 25 °C (controlled by the thermostat). The data was plotted against a reversible hydrogen electrode (RHE)

i $$E_{RHE}=E_{Hg/HgO}+0.059\times pH+E_{Hg/HgO}^{0}-iR$$

Considering the pH of 1 M aqueous KOH solution to be 13.89.^91^ During measurement, all CVs, LSVs, and CPs were reported after 90% *iR* correction. Before all the OER activity evaluation, all catalysts were activated to a minimum of 50 cycles at a scan rate of 10 mV/s in the potential region of 0.9 to 1.9 V_RHE_, until a stable CV was obtained. The activity was evaluated from the LSV curves under stirring conditions (400 rpm). A steady-state approach was used to derive the Tafel slopes by applying a constant potential for 180 s at an interval of 15 mV. The electrochemical impedance spectroscopy (EIS) was measured at 1.66 V (vs RHE). The amplitude was 10 mV, and the frequency range was from 10 kHz to 1 mHz. Impedance spectra were fitted by using an equivalent RC circuit model. The charge-transfer resistance (Rct) was calculated from the diameter of the semicircle in the Nyquist plot.

#### 4.8.2 Error Calculation

All the measurements (Tafel, CVs, LSV) were conducted a minimum of three times, and the error was calculated from the sample standard deviation.

ii $$S=\sqrt{\frac{\sum_{i=1}^{N}{\left(x_{i}-y\right)}^{2}}{N-1}}$$

where, *S* = sample standard deviation, *N* = the number of observations, *x*_*i*_ = the observed values of a sample item, and *y* = the mean value of the observations.

#### 4.8.3 Hybrid Water Electrolysis (Hydrogen Evolution Reaction Coupled with Ethylene Glycol Oxidation Reaction (EGOR))

The hybrid water electrolysis, i.e., hydrogen evolution reaction (HER) coupled with organic oxidation reaction (ethylene glycol oxidation in this case), was performed in a divided electrochemical cell employing a three-electrode configuration. The anodic and cathodic compartments were separated by an anion exchange membrane to minimize the product crossover and to keep distinct reaction environments. In the anodic chamber, ethylene glycol oxidation was performed on the self-supported electrode, while the cathodic chamber utilized a platinum wire electrode for the HER. Electrochemical measurements were carried out by using an electrolyte of 1 M ethylene glycol dissolved in 15 mL of 1 M KOH in the anodic chamber and 15 mL of 1 M KOH in the cathodic chamber. Cyclic voltammogram (CV) and chronoamperometric (at a cell potential of 1.38 V vs RHE) experiments were carried out, with continuous stirring at 400 rpm in the anodic compartment to ensure uniform mixing and efficient mass transport. We also performed the EGOR by varying the concentration of EG.

#### 4.8.4 ^1^H NMR Analysis of the Organic Compound

The reaction mixtures from oxidation experiments were analyzed using ^1^H Nuclear Magnetic Resonance (NMR) spectroscopy on a Bruker AV400 spectrometer. To prepare the NMR samples, a 150 μL aliquot of the reaction solution was mixed with 0.1 M dimethyl sulfoxide (DMSO, used as an internal standard) and 450 μL of deuterium oxide (D_2_O) as the solvent. The spectra were processed and visualized using MestReNova software. A prominent peak at 4.7 ppm was attributed to water (H_2_O) from the aqueous reaction mixture, serving as a reference for determining the chemical shifts of other proton signals.

#### 4.8.5 Calculation of the Faradaic Efficiency for the Oxidation of Ethylene Glycol

The Faradaic efficiency (FE) for EGOR was determined based on the moles of formate generated and the corresponding charge passed during the reaction. The product identification and quantification were carried out using ^1^H NMR spectroscopy with DMSO as an internal standard of known concentration. The FE for formate production was calculated by using the following formula:

The FEs of liquid products were calculated from the NMR peak area.

$${FE}_{liquid}=\frac{mole\;of\;the\;liquid\;product\;formed\times F\times n_{e}}{total\;charge\;passed\;\left(Q\right)}\times 100\%$$

where *F* is the Faraday constant (96,485 C mol^–1^), *n*_e_ is the number of electron transfer for each product formation, *n*_e_ = 3 for formate, *n*_e_ = 4 for glycolate.

## Supplementary Material



## Notes

Additional experimental methods; detailed experimental protocols; and spectroscopic and microscopic characterizations of the catalysts (PDF)

## References

[1] de
Kleijne K., Huijbregts M. A. J., Knobloch F., van Zelm R., Hilbers J. P., de Coninck H., Hanssen S. V. (2024). Worldwide greenhouse
gas emissions of green hydrogen production and transport. Nat. Energy.

[2] Anantharaj S., Noda S., Jothi V. R., Yi S., Driess M., Menezes P. W. (2021). Strategies and Perspectives to Catch
the Missing Pieces
in Energy-Efficient Hydrogen Evolution Reaction in Alkaline Media. Angew. Chem., Int. Ed..

[3] Roy S., Bagchi D., Dheer L., Sarma S. C., Rajaji V., Narayana C., Waghmare U. V., Peter S. C. (2021). Mechanistic insights
into the promotional effect of Ni substitution in non-noble metal
carbides for highly enhanced water splitting. Appl. Catal. B: Environ..

[4] Bagchi D., Walter C., Tandava V. S. R. K., Cobos-Becerra Y. L., Fletcher J. C. Q., Fischer N., Sontheimer T., Menezes P. W. (2026). Electrochemical Carbon Dioxide Reduction
to Methanol
on Copper-Based Catalysts: Mechanistic Insights and Industrial Prospects. Adv. Mater..

[5] Mondal S., Sarkar S., Bagchi D., Das T., Das R., Singh A. K., Prasanna P. K., Vinod C. P., Chakraborty S., Peter S. C. (2022). Morphology-Tuned Pt3Ge Accelerates
Water Dissociation
to Industrial-Standard Hydrogen Production over a wide pH Range. Adv. Mater..

[6] Menezes P. W., Indra A., Das C., Walter C., Göbel C., Gutkin V., Schmeiβer D., Driess M. (2017). Uncovering the Nature
of Active Species of Nickel Phosphide Catalysts in High-Performance
Electrochemical Overall Water Splitting. ACS
Catal..

[7] Chen Z., Wu R., Liu Y., Ha Y., Guo Y., Sun D., Liu M., Fang F. (2018). Ultrafine
Co Nanoparticles Encapsulated in Carbon-Nanotubes-Grafted
Graphene Sheets as Advanced Electrocatalysts for the Hydrogen Evolution
Reaction. Adv. Mater..

[8] Yang H., Guo P., Wang R., Chen Z., Xu H., Pan H., Sun D., Fang F., Wu R. (2022). Sequential Phase Conversion-Induced
Phosphides Heteronanorod Arrays for Superior Hydrogen Evolution Performance
to Pt in Wide pH Media. Adv. Mater..

[9] Li W., Liu J., Guo P., Li H., Fei B., Guo Y., Pan H., Sun D., Fang F., Wu R. (2021). Co/CoP Heterojunction
on Hierarchically Ordered Porous Carbon as a Highly Efficient Electrocatalyst
for Hydrogen and Oxygen Evolution. Adv. Energy
Mater..

[10] McCrory C. C. L., Jung S., Ferrer I. M., Chatman S. M., Peters J. C., Jaramillo T. F. (2015). Benchmarking Hydrogen Evolving Reaction
and Oxygen
Evolving Reaction Electrocatalysts for Solar Water Splitting Devices. J. Am. Chem. Soc..

[11] Bagchi D., Phukan N., Sarkar S., Das R., Ray B., Bellare P., Ravishankar N., Peter S. C. (2021). Ultralow non-noble
metal loaded MOF derived bi-functional electrocatalysts for the oxygen
evolution and reduction reactions. J. Mater.
Chem. A.

[12] Li S., Liu T., Zhang W., Wang M., Zhang H., Qin C., Zhang L., Chen Y., Jiang S., Liu D., Liu X., Wang H., Luo Q., Ding T., Yao T. (2024). Highly efficient
anion exchange membrane water electrolyzers via chromium-doped amorphous
electrocatalysts. Nat. Commun..

[13] Mondal S., Riyaz M., Bagchi D., Dutta N., Singh A. K., Vinod C. P., Peter S. C. (2023). Distortion-Induced
Interfacial Charge
Transfer at Single Cobalt Atom Secured on Ordered Intermetallic Surface
Enhances Pure Oxygen Production. ACS Nano.

[14] Panda C., Menezes P. W., Walter C., Yao S., Miehlich M. E., Gutkin V., Meyer K., Driess M. (2017). From a Molecular
2Fe-2Se
Precursor to a Highly Efficient Iron Diselenide Electrocatalyst for
Overall Water Splitting. Angew. Chem., Int.
Ed..

[15] Liu D., Guo P., Wang Q., Ding X., He Y., Zhou J., Sun D., Pan H., Wu R. (2025). Local Proton-Mediated Synthesis of
a High-Entropy Borate Library. Adv. Mater..

[16] Singh A. K., Bagchi D., Sarkar S., Sarma S. C., Mumbaraddi D., Ramarao S. D., Peter S. C. (2022). Optimized Metal Deficiency-Induced
Operando Phase Transformation Enhances Charge Polarization Promoting
Hydrogen Evolution Reaction. Chem. Mater..

[17] Mondal S., Sarkar S., Kediya S., Riyaz M., Singh A., Das S., Bagchi D., Burman R., Dutta N., Singh A. K., Radhakrishnan M., Peter S. C. (2026). Unravelling the Growth Mechanism
of Local Entropy Tailored Intermetallic Pd3Ni Exhibiting Tetrafunctional
Activity in a Water Electrolyzer and Fuel Cell. ACS Nano.

[18] Bharadwaj B., Gates T., Rose S., Antriyandarti E., Praveena S. M., Oranu C. O., Borthakur M., Dhungana P. K., Shazly A., De-la-Torre G. E., Allison A. L., Abeywardhana D. M. Y., Mabaso S., Adom P. K., Banga M., Dlamini W., Kabera T., Bohlmann J., Areeprasert C., Veettil B. K., Dieudonné Shukuru W., Nkhwanana N., Kammwamba A., Rai R. K., Conteh B., Erasmus V. N., Agbere S., Phonhalath K., Njoroge H., Glenn D., Ishuga E., Mugisho G. M., Moolla R., Hounnou F. E., Guloba M. M., Damiran U., Vuthaluru H., Mulagetta Y., Jeuland M., Gates I. D., Ashworth P. (2026). Prevalence of plastic waste as a household fuel in
low-income communities of the Global South. Nat. Commun..

[19] Tan A. F. J., Yu S., Wang C., Yeoh G. H., Teoh W. Y., Yip A. C. K. (2024). Reimagining plastics
waste as energy solutions: challenges
and opportunities. npj Mater. Sustainability.

[20] Plastics give and plastics take. Nat. Rev. Mater. 2022, 7, 67–. 10.1038/s41578-022-00419-y

[21] Mondal I., Ghosh S., Mebs S., An N., Yang R., Ahamad T., Zebger I., Reith L., Dau H., Driess M., Menezes P. W. (2026). Substrate-induced redox reconstruction
of nickel sites in organic oxidation electrocatalysis. Chem Catal..

[22] Geyer R., Jambeck J. R., Law K. L. (2017). Production, use,
and fate of all
plastics ever made. Sci. Adv..

[23] MacLeod M., Arp H. P. H., Tekman M. B., Jahnke A. (2021). The global threat from
plastic pollution. Science.

[24] Wang Y., Liu F., Chen J., Tse E. C. M., Shi R., Chen Y. (2025). Scale-up upcycling
of waste polyethylene terephthalate plastics to biodegradable polyglycolic
acid plastics. Nat. Commun..

[25] Singh N., Walker T. R. (2024). Plastic recycling: A panacea or environmental pollution
problem. npj Mater. Sustainability.

[26] Zhao G., Lin J., Lu M., Li L., Xu P., Liu X., Chen L. (2024). Potential cycling boosts the electrochemical
conversion of polyethylene
terephthalate-derived alcohol into valuable chemicals. Nat. Commun..

[27] Sun J., Shi B., Dai S., Chu L., Wang H., Huang M. (2025). Promoted *OH
Adsorption Facilitates C–C Bond Cleavage for Efficient Electrochemical
Upcycling of Polyethylene Terephthalate. ACS
Catal..

[28] Dasgupta B., Bagchi D., Sontheimer T., Driess M., Menezes P. W. (2025). Dynamics
in electrochemical organic oxidation reactions from in situ and operando
techniques. Nat. Rev. Chem..

[29] Li J., Li L., Ma X., Han X., Xing C., Qi X., He R., Arbiol J., Pan H., Zhao J., Deng J., Zhang Y., Yang Y., Cabot A. (2023). Selective
Ethylene
Glycol Oxidation to Formate on Nickel Selenide with Simultaneous Evolution
of Hydrogen. Adv. Sci..

[30] Ghosh S., Bagchi D., Mondal I., Sontheimer T., Jagadeesh R. V., Menezes P. W. (2024). Deciphering the Role of Nickel in
Electrochemical Organic Oxidation Reactions. Adv. Energy Mater..

[31] Zhou H., Ren Y., Li Z., Xu M., Wang Y., Ge R., Kong X., Zheng L., Duan H. (2021). Electrocatalytic upcycling
of polyethylene terephthalate to commodity chemicals and H2 fuel. Nat. Commun..

[32] Si D., Xiong B., Chen L., Shi J. (2021). Highly selective and
efficient electrocatalytic synthesis of glycolic acid in coupling
with hydrogen evolution. Chem Catal..

[33] Yang H., Vijaykumar G., Chen Z., Hausmann J. N., Mondal I., Ghosh S., Nicolaus V. C. J., Laun K., Zebger I., Driess M., Menezes P. W. (2023). In Situ Reconstruction of Helical
Iron Borophosphate Precatalyst toward Durable Industrial Alkaline
Water Electrolysis and Selective Oxidation of Alcohols. Adv. Funct. Mater..

[34] Wu X., Wang Y., Wu Z.-S. (2022). Design
principle of electrocatalysts
for the electrooxidation of organics. Chem.

[35] Deng K., Lian Z., Wang W., Yu J., Yu H., Wang Z., Xu Y., Wang L., Wang H. (2024). Lattice Strain
and Charge Redistribution of Pt Cluster/Ir Metallene Heterostructure
for Ethylene Glycol to Glycolic Acid Conversion Coupled with Hydrogen
Production. Small.

[36] Wang N., Li X., Hu M.-K., Wei W., Zhou S.-H., Wu X.-T., Zhu Q.-L. (2022). Ordered macroporous superstructure of bifunctional
cobalt phosphide with heteroatomic modification for paired hydrogen
production and polyethylene terephthalate plastic recycling. Appl. Catal. B: Environ..

[37] Liu F., Gao X., Shi R., Guo Z., Tse E. C. M., Chen Y. (2023). Concerted
and Selective Electrooxidation of Polyethylene-Terephthalate-Derived
Alcohol to Glycolic Acid at an Industry-Level Current Density over
a Pd–Ni­(OH)­2 Catalyst. Angew. Chem.,
Int. Ed..

[38] Roberts C. C., Camasso N. M., Bowes E. G., Sanford M. S. (2019). Impact of Oxidation
State on Reactivity and Selectivity Differences between Nickel­(III)
and Nickel­(IV) Alkyl Complexes. Angew. Chem.,
Int. Ed..

[39] Fan K., Chen H., Ji Y., Huang H., Claesson P. M., Daniel Q., Philippe B., Rensmo H., Li F., Luo Y., Sun L. (2016). Nickel–vanadium
monolayer double hydroxide for
efficient electrochemical water oxidation. Nat.
Commun..

[40] Wang Y., Chen C., Xiong X., Skaanvik S. A., Zhang Y., Bøjesen E. D., Wang Z., Liu W., Dong M. (2024). In Situ Tracking
of Water Oxidation Generated Nanoscale Dynamics in Layered Double
Hydroxides Nanosheets. J. Am. Chem. Soc..

[41] Scarabelli L., Sun M., Zhuo X., Yoo S., Millstone J. E., Jones M. R., Liz-Marzán L.
M. (2023). Plate-Like Colloidal
Metal Nanoparticles. Chem. Rev..

[42] Lee J. M., No Y.-S., Kim S., Park H.-G., Park W. I. (2015). Strong
interactive growth behaviours in solution-phase synthesis of three-dimensional
metal oxide nanostructures. Nat. Commun..

[43] Terlemezoglu M., Surucu O., Isik M., Gasanly N. M., Parlak M. (2021). Temperature-dependent
optical characteristics of sputtered NiO thin films. Appl. Phys. A: Mater. Sci. Process..

[44] Ghosh S., Dasgupta B., Kalra S., Ashton M. L. P., Yang R., Kueppers C. J., Gok S., Alonso E. G., Schmidt J., Laun K., Zebger I., Walter C., Driess M., Menezes P. W. (2023). Evolution of Carbonate-Intercalated γ-NiOOH from
a Molecularly Derived Nickel Sulfide (Pre)­Catalyst for Efficient Water
and Selective Organic Oxidation. Small.

[45] Li H. B., Yu M. H., Wang F. X., Liu P., Liang Y., Xiao J., Wang C. X., Tong Y. X., Yang G. W. (2013). Amorphous
nickel hydroxide nanospheres with ultrahigh capacitance and energy
density as electrochemical pseudocapacitor materials. Nat. Commun..

[46] Bazan-Aguilar A., García G., Pastor E., Rodríguez J. L., Baena-Moncada A. M. (2023). In-situ
spectroelectrochemical study of highly active
Ni-based foam electrocatalysts for hydrogen evolution reaction. Appl. Catal. B: Environ..

[47] Shinagawa T., Kotobuki N., Ohtaka A. (2022). Oriented growth
of stacking α-cobalt
hydroxide salt continuous films and their topotactic-like transformation
to oriented mesoporous films of Co3O4 and CoO. Nanoscale Adv..

[48] Liu M., Min K.-A., Han B., Lee L. Y. S. (2021). Interfacing or
Doping? Role of Ce in Highly Promoted Water Oxidation of NiFe-Layered
Double Hydroxide. Adv. Energy Mater..

[49] Zhu B., Dong B., Wang F., Yang Q., He Y., Zhang C., Jin P., Feng L. (2023). Unraveling a bifunctional
mechanism for methanol-to-formate electro-oxidation on nickel-based
hydroxides. Nat. Commun..

[50] Chala S. A., Tsai M.-C., Su W.-N., Ibrahim K. B., Thirumalraj B., Chan T.-S., Lee J.-F., Dai H., Hwang B.-J. (2020). Hierarchical
3D Architectured Ag Nanowires Shelled with NiMn-Layered Double Hydroxide
as an Efficient Bifunctional Oxygen Electrocatalyst. ACS Nano.

[51] Friebel D., Louie M. W., Bajdich M., Sanwald K. E., Cai Y., Wise A. M., Cheng M.-J., Sokaras D., Weng T.-C., Alonso-Mori R., Davis R. C., Bargar J. R., Nørskov J. K., Nilsson A., Bell A. T. (2015). Identification of Highly Active Fe
Sites in (Ni,Fe)­OOH for Electrocatalytic Water Splitting. J. Am. Chem. Soc..

[52] Biesinger M. C., Payne B. P., Grosvenor A. P., Lau L. W. M., Gerson A. R., Smart R. S. C. (2011). Resolving
surface chemical states in XPS analysis of
first row transition metals, oxides and hydroxides: Cr, Mn, Fe, Co
and Ni. Appl. Surf. Sci..

[53] Thenuwara A. C., Attanayake N. H., Yu J., Perdew J. P., Elzinga E. J., Yan Q., Strongin D. R. (2018). Cobalt Intercalated
Layered NiFe Double Hydroxides
for the Oxygen Evolution Reaction. J. Phys.
Chem. B.

[54] Bagchi D., Sarkar S., Singh A. K., Vinod C. P., Peter S. C. (2022). Potential-
and Time-Dependent Dynamic Nature of an Oxide-Derived PdIn Nanocatalyst
during Electrochemical CO2 Reduction. ACS Nano.

[55] Dionigi F., Zeng Z., Sinev I., Merzdorf T., Deshpande S., Lopez M. B., Kunze S., Zegkinoglou I., Sarodnik H., Fan D., Bergmann A., Drnec J., Araujo J. F. d., Gliech M., Teschner D., Zhu J., Li W.-X., Greeley J., Cuenya B. R., Strasser P. (2020). In-situ structure
and catalytic mechanism of NiFe and CoFe layered double hydroxides
during oxygen evolution. Nat. Commun..

[56] Chen Z., Yang H., Hausmann J. N., Mebs S., Hlukhyy V., Dau H., Driess M., Menezes P. W. (2025). Ba–Ni–Ge
Clathrate
Transformation Maximizes Active Site Utilization of Nickel for Enhanced
Oxygen Evolution Performance. Angew. Chem.,
Int. Ed..

[57] Yang H., Driess M., Menezes P. W. (2021). Self-Supported
Electrocatalysts for
Practical Water Electrolysis. Adv. Energy Mater..

[58] Quan L., Jiang H., Mei G., Sun Y., You B. (2024). Bifunctional
Electrocatalysts for Overall and Hybrid Water Splitting. Chem. Rev..

[59] Swierk J. R., Klaus S., Trotochaud L., Bell A. T., Tilley T. D. (2015). Electrochemical
Study of the Energetics of the Oxygen Evolution Reaction at Nickel
Iron (Oxy)­Hydroxide Catalysts. J. Phys. Chem.
C.

[60] Mondal I., Hausmann J. N., Vijaykumar G., Mebs S., Dau H., Driess M., Menezes P. W. (2022). Nanostructured
Intermetallic Nickel
Silicide (Pre)­Catalyst for Anodic Oxygen Evolution Reaction and Selective
Dehydrogenation of Primary Amines. Adv. Energy
Mater..

[61] van
der Heijden O., Park S., Vos R. E., Eggebeen J. J. J., Koper M. T. M. (2024). Tafel Slope Plot as a Tool to Analyze Electrocatalytic
Reactions. ACS Energy Lett..

[62] Anantharaj S., Noda S., Driess M., Menezes P. W. (2021). The Pitfalls
of
Using Potentiodynamic Polarization Curves for Tafel Analysis in Electrocatalytic
Water Splitting. ACS Energy Lett..

[63] Burger J. P., MacLachlan D. S., Mailfert R., Souffaché B. (1975). Electrical
resistivity of Pd—Hx: I. Residual resistivity. Solid State Commun..

[64] Hall D. S., Lockwood D. J., Bock C., MacDougall B. R. (2015). Nickel
hydroxides and related materials: a review of their structures, synthesis
and properties. Proc. R. Soc. A.

[65] Gong M., Li Y., Wang H., Liang Y., Wu J. Z., Zhou J., Wang J., Regier T., Wei F., Dai H. (2013). An Advanced
Ni–Fe Layered Double Hydroxide Electrocatalyst for Water Oxidation. J. Am. Chem. Soc..

[66] Laan P. C.
M., de Zwart F. J., Wilson E. M., Troglia A., Lugier O. C. M., Geels N. J., Bliem R., Reek J. N. H., de
Bruin B., Rothenberg G., Yan N. (2023). Understanding the Oxidative
Properties of Nickel Oxyhydroxide in Alcohol Oxidation Reactions. ACS Catal..

[67] Kuai C., Xi C., Hu A., Zhang Y., Xu Z., Nordlund D., Sun C.-J., Cadigan C. A., Richards R. M., Li L., Dong C.-K., Du X.-W., Lin F. (2021). Revealing the Dynamics
and Roles of Iron Incorporation in Nickel Hydroxide Water Oxidation
Catalysts. J. Am. Chem. Soc..

[68] Hu Y., Chao T., Dou Y., Xiong Y., Liu X., Wang D. (2025). Isolated Metal Centers Activate Small Molecule Electrooxidation:
Mechanisms and Applications. Adv. Mater..

[69] Jiang X., Ma X., Yang Y., Liu Y., Liu Y., Zhao L., Wang P., Zhang Y., Lin Y., Wei Y. (2024). Enhancing
the Electrocatalytic Oxidation of 5-Hydroxymethylfurfural Through
Cascade Structure Tuning for Highly Stable Biomass Upgrading. Nano-Micro Lett..

[70] Tian B., Wang F., Ran P., Dai L., Lv Y., Sun Y., Mu Z., Sun Y., Tang L., Goddard W. A., Ding M. (2024). Parameterization and
quantification of two key operando physio-chemical
descriptors for water-assisted electro-catalytic organic oxidation. Nat. Commun..

[71] Li Z., Jiang K., Yang Y., Pian Y., Liu H., Zheng Z., Wang X., Jana S., Li J., Ma Z., Qiao X., Zou X., Ma X., Zhang B., Chu H., Wu Y. A. (2025). Pd@PdBOx
heterostructure with strong electronic interaction
to promote C–H bond activation for glycerol oxidation reaction. Sci. Adv..

[72] Antolini E. (2009). Palladium
in fuel cell catalysis. Energy Environ. Sci..

[73] Bagchi D., Riyaz M., Dutta N., Chawla G., R Churipard S., Kumar Singh A., C Peter S. (2024). Operando Investigation of the Origin
of C–C Coupling in Electrochemical CO2 Reduction Upon Releasing
Bonding Strength, Structural Ordering in Pd–Cu Catalyst. Adv. Energy Mater..

[74] Liu Q., Ye F., Guan K., Yang Y., Dong H., Wu Y., Tang Z., Hu L. (2023). MnAl Layered Double Hydroxides: A
Robust Host for Aqueous Ammonium-Ion Storage with Stable Plateau and
High Capacity. Adv. Energy Mater..

[75] Maxwell D. S., Kendrick I., Mukerjee S. (2024). Operando Raman Spectroscopy Reveals
Degradation Byproducts from Ionomer Oxidation in Anion Exchange Membrane
Water Electrolyzers. J. Am. Chem. Soc..

[76] Yeo B. S., Bell A. T. (2012). In Situ Raman Study of Nickel Oxide
and Gold-Supported
Nickel Oxide Catalysts for the Electrochemical Evolution of Oxygen. J. Phys. Chem. C.

[77] Dasgupta B., Ghosh S., Walter C., Budde M. S., Marquardt G. J., Chen H.-H., Breithaupt M. G. M., Yilmaz T., Garmatter C., Ahamad T., Zebger I., Driess M., Menezes P. W. (2024). A soft
molecular single-source precursor approach to synthesize a nanostructured
Co9S8 (pre)­catalyst for efficient water oxidation and biomass valorization. J. Mater. Chem. A.

[78] Mondal I., Menezes P. V., Laun K., Diemant T., Al-Shakran M., Zebger I., Jacob T., Driess M., Menezes P. W. (2023). In-Liquid
Plasma-Mediated Manganese Oxide Electrocatalysts for Quasi-Industrial
Water Oxidation and Selective Dehydrogenation. ACS Nano.

[79] Novikov V. S., Liubimovskii S. O., Kuznetsov S. M., Mel’nik N. N., Sagitova E. A., Aiyyzhy K. O., Ivchenko P. V., Kuzmin V. V., Gudkov S. V., Moskovskiy M. N., Nikolaeva G. Y. (2025). Raman analysis
of aqueous solutions of ethylene glycol and 1,3-propylene glycol:
Fundamental and applied aspects. Spectrochim.
Acta, Part A.

[80] Liubimovskii S. O., Novikov V. S., Ustynyuk L. Y., Ivchenko P. V., Prokhorov K. A., Kuzmin V. V., Sagitova E. A., Godyaeva M. M., Gudkov S. V., Darvin M. E., Nikolaeva G. Y. (2023). Raman structural
study of ethylene
glycol and 1,3-propylene glycol aqueous solutions. Spectrochim. Acta, Part A Mol. Biomol. Spectrosc..

[81] Krishnan K., Krishnan R. S. (1966). Raman and infrared spectra of ethylene
glycol. Proc. Ind. Acad. Sci..

[82] Wang L., Meng H., Shen P. K., Bianchini C., Vizza F., Wei Z. J. P. C. C. P. (2011). In situ FTIR spectroelectrochemical
study on the mechanism of ethylene glycol electrocatalytic oxidation
at a Pd electrode. Phys. Chem. Chem. Phys..

[83] Guo H., Shi Q., Xu Y., Yue G., Liang Z., Yue C., Ye S., Liu Q., Wei Q., Yang D., Yang W. (2026). Direct activation
of layered double hydroxides for efficient and durable water splitting
at high current densities. Appl. Catal. B Environ..

[84] Mohaček-Grošev V., Šoštarić V., Maksimović A. J. S. a. P. A. (2015). Molecular; spectroscopy, b., Raman
spectroscopic evidence of low
temperature stability of D,L-glycolic and L-(+)-lactic acid crystals. Spectrochim. Acta, Part A Mol. Biomol. Spectrosc..

[85] Hammer B., Nørskov J. K. (2000). Theoretical surface science and catalysis—calculations
and concepts. Adv. Catal..

[86] Suntivich J., May K. J., Gasteiger H. A., Goodenough J. B., Shao-Horn Y. (2011). A Perovskite Oxide Optimized for
Oxygen Evolution Catalysis
from Molecular Orbital Principles. Science.

[87] Liu D., Lv Q., Zheng D., Zhou C., Chen S., Zhang K., Han S., Huang H.-Z., Zhang Y., Chen L. (2025). Strategic Design of
Ethanol Oxidation Catalysts: From Active Metal Selection to Mechanistic
Insights and Performance Engineering. Nanomaterials.

[88] Menezes P. W., Yao S., Beltrán-Suito R., Hausmann J. N., Menezes P. V., Driess M. (2021). Facile Access to an Active γ-NiOOH Electrocatalyst
for Durable Water Oxidation Derived From an Intermetallic Nickel Germanide
Precursor. Angew. Chem., Int. Ed..

[89] Menezes P. W., Panda C., Garai S., Walter C., Guiet A., Driess M. (2018). Structurally Ordered Intermetallic
Cobalt Stannide
Nanocrystals for High-Performance Electrocatalytic Overall Water-Splitting. Angew. Chem., Int. Ed..

[90] Bozza F., Polini R., Traversa E. (2008). Electrophoretic Deposition of Dense
Sr- and Mg-Doped LaGaO3 Electrolyte Films on Porous La-Doped Ceria
for Intermediate Temperature Solid Oxide Fuel Cells. Fuel Cells.

[91] Hausmann J. N., Traynor B., Myers R. J., Driess M., Menezes P. W. (2021). The pH
of Aqueous NaOH/KOH Solutions: A Critical and Non-trivial Parameter
for Electrocatalysis. ACS Energy Lett..

