# Supplementary material for: Rational Engineering of Self-Supported Catalysts for High-Performance Electrochemical Oxygen Evolution and Ethylene Glycol Oxidation
Source: Chem Mater. 2026 May 13;38(10):5098–111. doi: 10.1021/acs.chemmater.6c00346 (PMC13220160; doi:10.1021/acs.chemmater.6c00346)
Supplement: Supplementary file 1 [file cm6c00346_si_001.pdf]

Supporting information

Rational Engineering of Self-Supported Catalysts for High-Performance Electrochemical Oxygen Evolution and Ethylene Glycol Oxidation

Debabrata Bagchi,[a] Gowtham Kenguva,[a] Suptish Ghosh,[b] Zishuo Zhang,[c] Sagie Katz,[c] Johannes Schmidt,[d] Ingo Zebger,[c] Tobias Sontheimer,[e] Prashanth W. Menezes[a,*]

[a]*Department of Materials Chemistry for Catalysis, Helmholtz-Zentrum Berlin für Materialien GmbH, Hahn-Meitner-Platz 1, Berlin, Germany.*

[b]*Department of Chemistry: Metalorganics and Inorganic Materials, Technische Universität Berlin, Straße des 17 Juni 135, Sekr. C2, 10623 Berlin, Germany.*

[c]*Department of Chemistry: Biophysical Chemistry, Technische Universität Berlin, Straße des 17 Juni 115, Secr. PC14, 10623 Berlin, Germany.*

[d]*Functional Materials, Institute of Chemistry, Faculty II Mathematics and Natural Sciences, Technische Universität Berlin, 10623 Berlin, Germany.*

[e]*Strategy Department of Energy and Information, Helmholtz-Zentrum Berlin für Materialien und Energie GmbH, Hahn-Meitner-Platz 1, Berlin, Germany.*

Figures

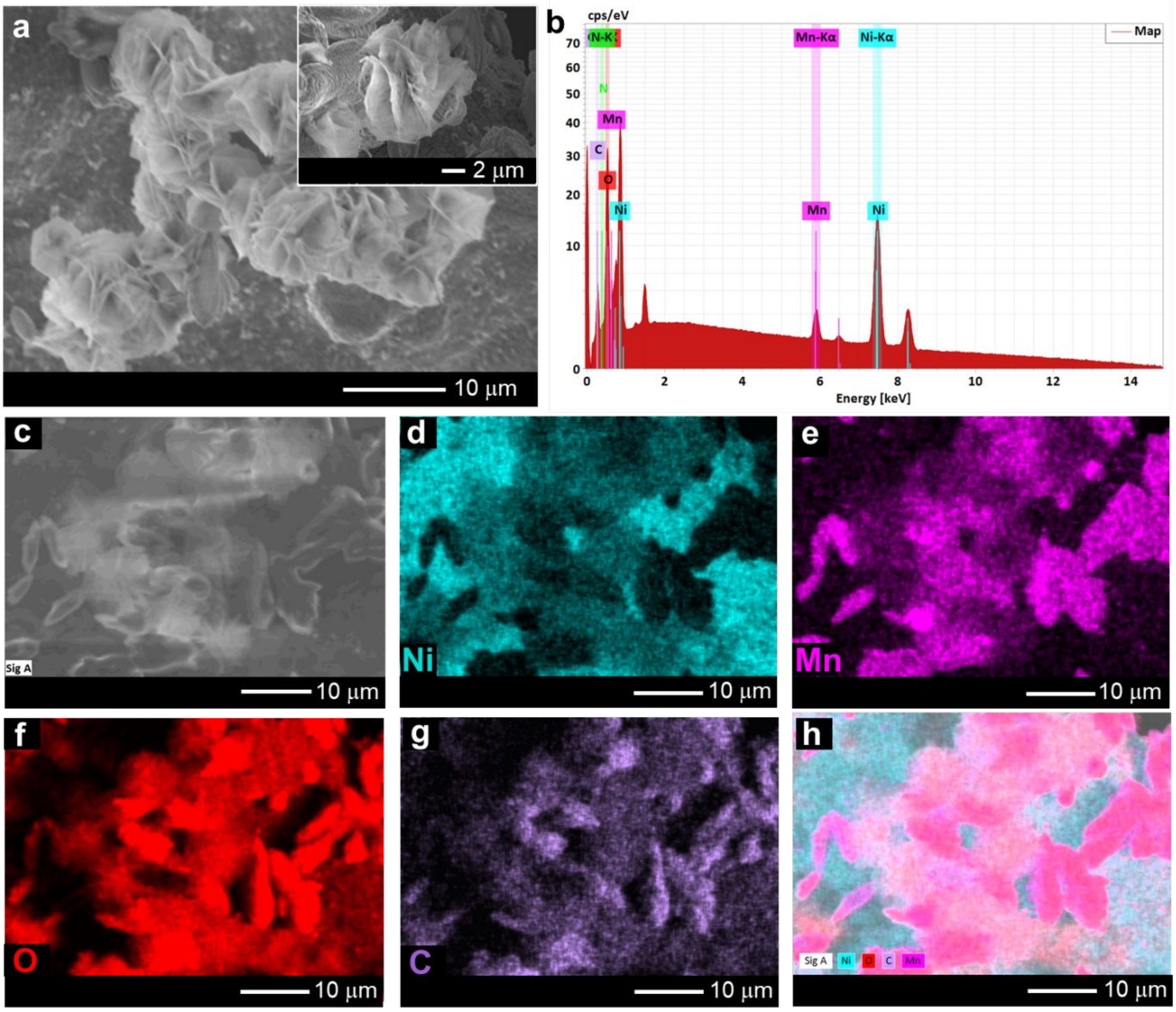

Figure S1. (a) SEM images of the hydrothermally grown Mn-NiO_xH_y/NF self-supported electrode and corresponding (b) EDX spectra confirming the presence of different elements in the sample. (c-h) The SEM-EDX elemental mapping for (d) Ni, (e) Mn, (f) O, (g) C and (h) combined elemental overlay, demonstrating homogeneous elemental distribution of the constituent elements across the catalyst surface.

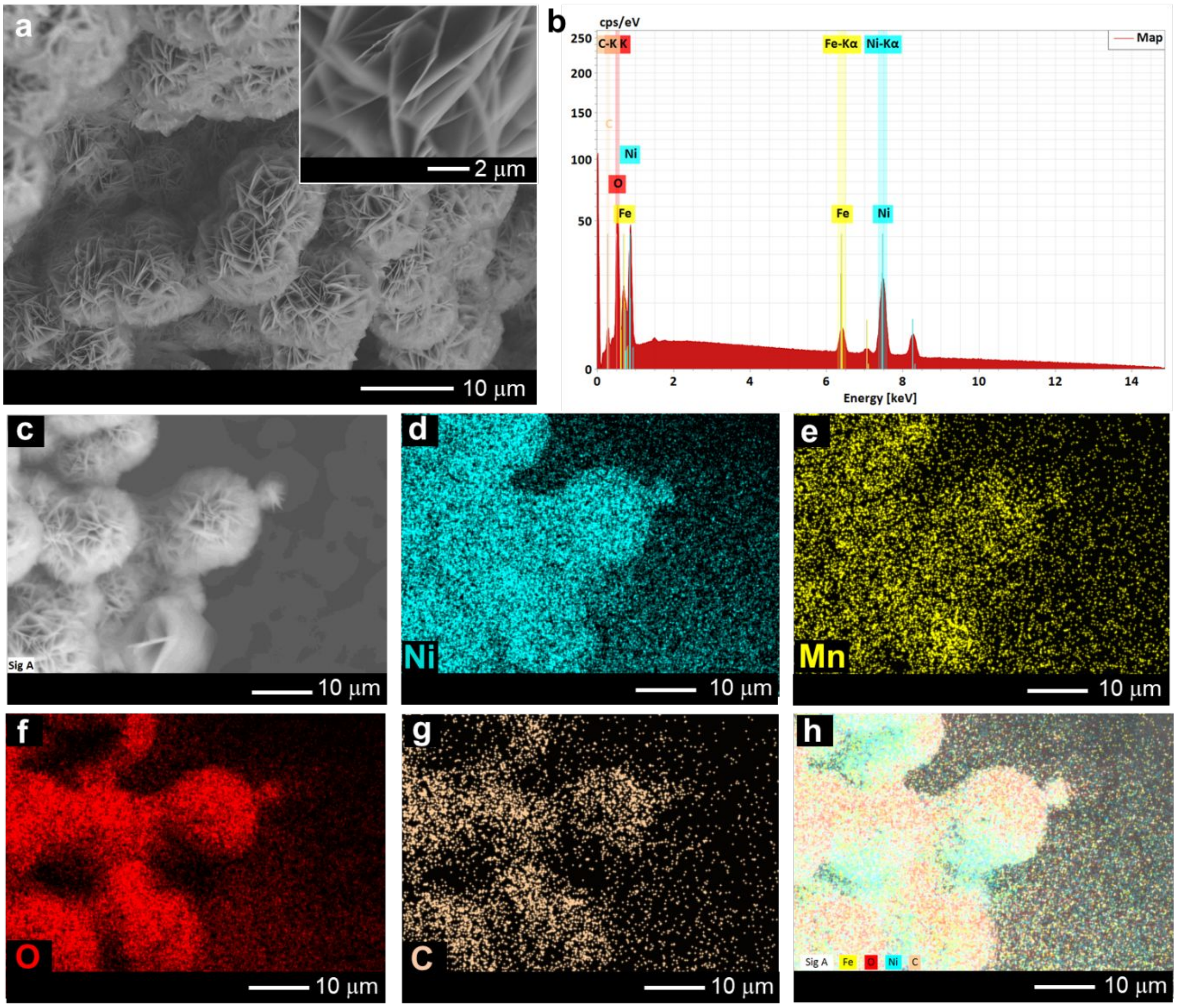

Figure S2. (a) SEM images of the hydrothermally grown Fe-NiO_xH_y/NF self-supported electrode and corresponding (b) EDX spectra confirming the presence of different elements in the sample. (c-h) The SEM-EDX elemental mapping for (d) Ni, (e) Fe, (f) O, (g) C and (h) combined elemental overlay, demonstrating homogeneous elemental distribution of the constituent elements across the catalyst surface.

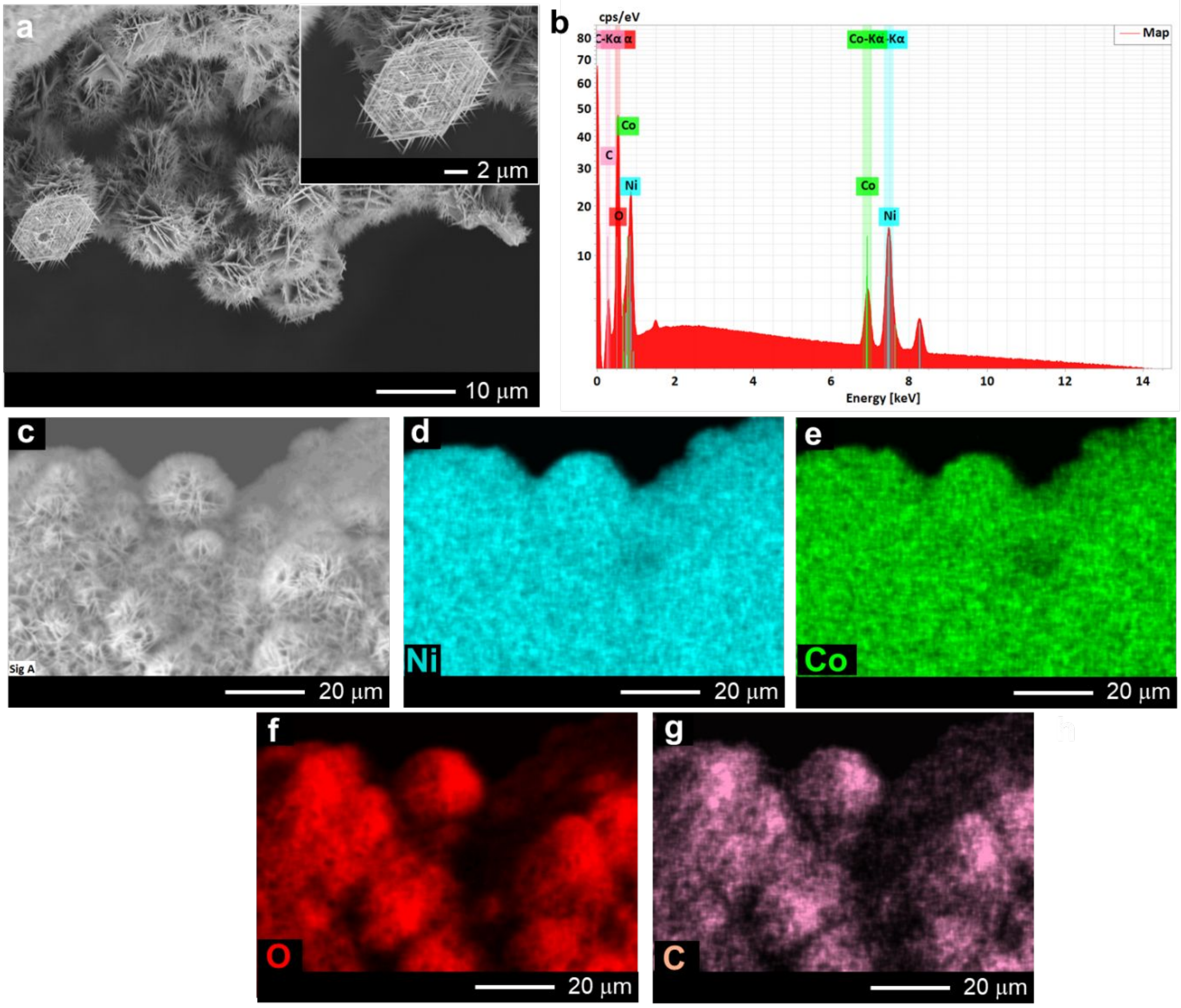

Figure S3. (a) SEM images of the hydrothermally grown Co-NiO_xH_y/NF self-supported electrode and corresponding (b) EDX spectra confirming the presence of different elements in the sample. (c-g) The SEM-EDX elemental mapping for (d) Ni, (e) Co, (f) O, and (g) C, demonstrating homogeneous elemental distribution of the constituent elements across the catalyst surface.

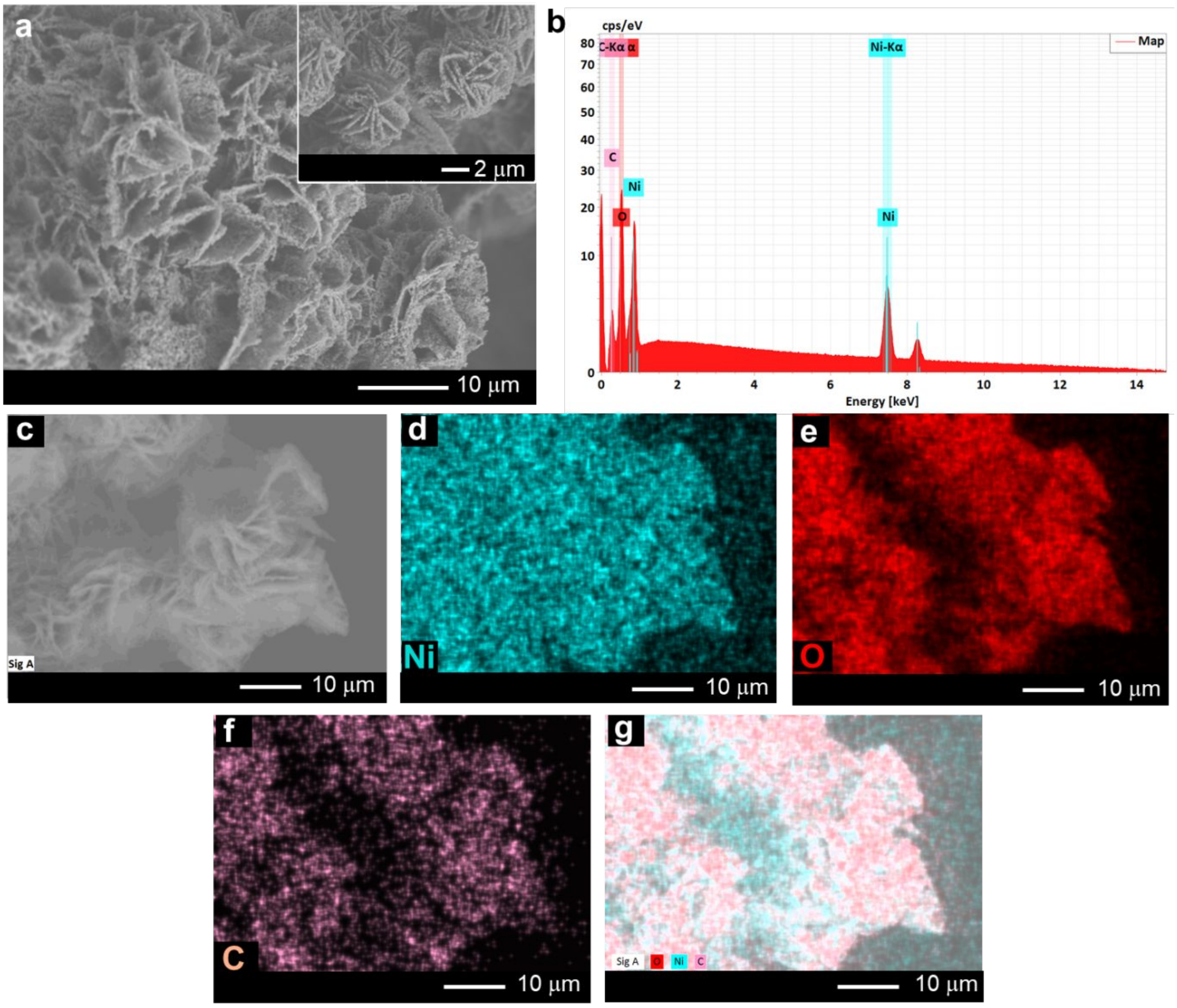

Figure S4. (a) SEM images of the hydrothermally grown Ni-NiO_xH_y/NF self-supported electrode and corresponding (b) EDX spectra confirming the presence of different elements in the sample. (c-g) The SEM-EDX elemental mapping for (d) Ni, (e) O, (f) C, and (g) combined elemental overlay, demonstrating homogeneous elemental distribution of the constituent elements across the catalyst surface.

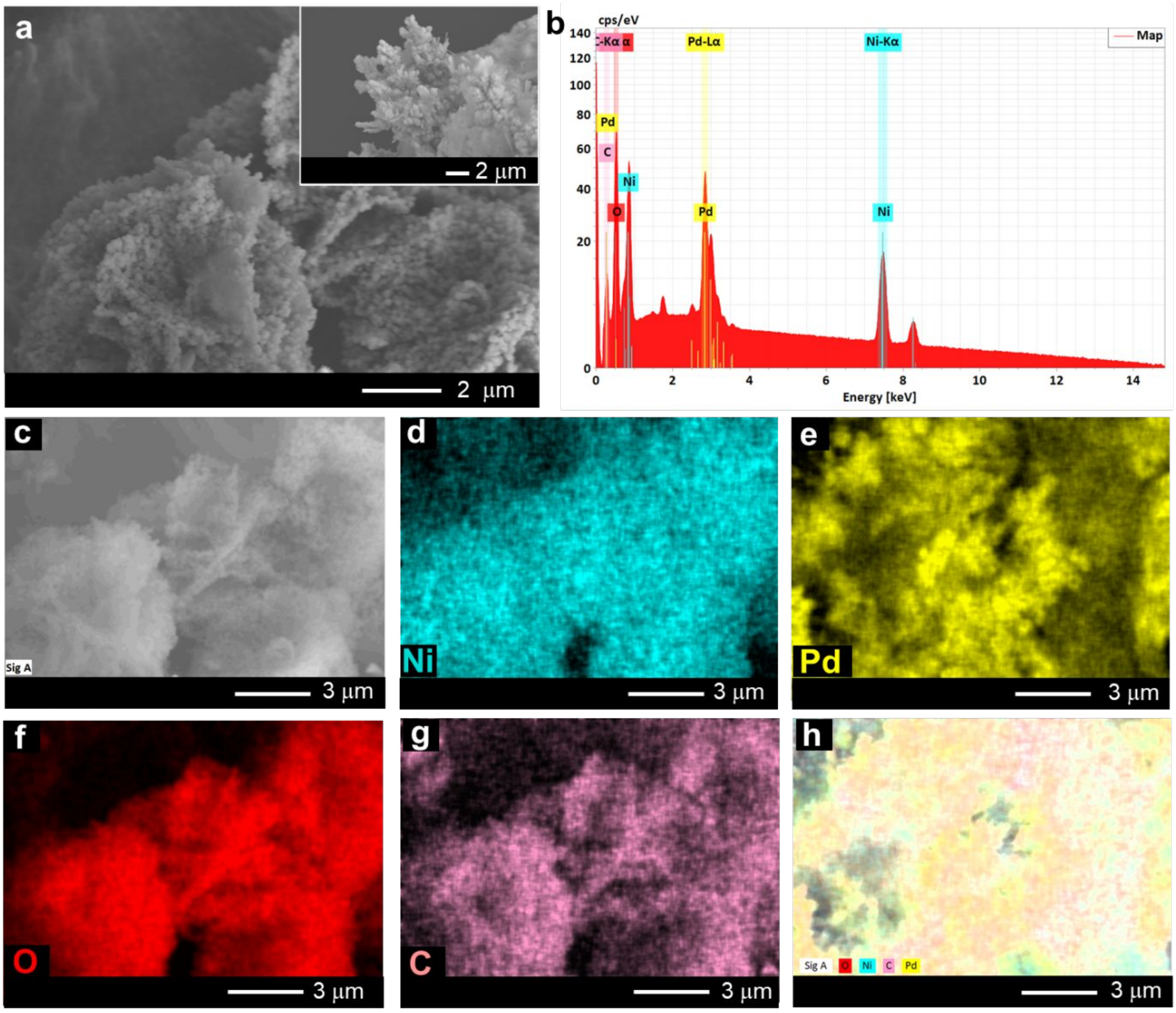

Figure S5. (a) SEM images of the hydrothermally grown Pd-NiO_xH_y/NF self-supported electrode and corresponding (b) EDX spectra confirming the presence of different elements in the sample. (c-h) The SEM-EDX elemental mapping for (d) Ni, (e) Pd, (f) O, (g) C and (h) combined elemental overlay, demonstrating homogeneous elemental distribution of the constituent elements across the catalyst surface.

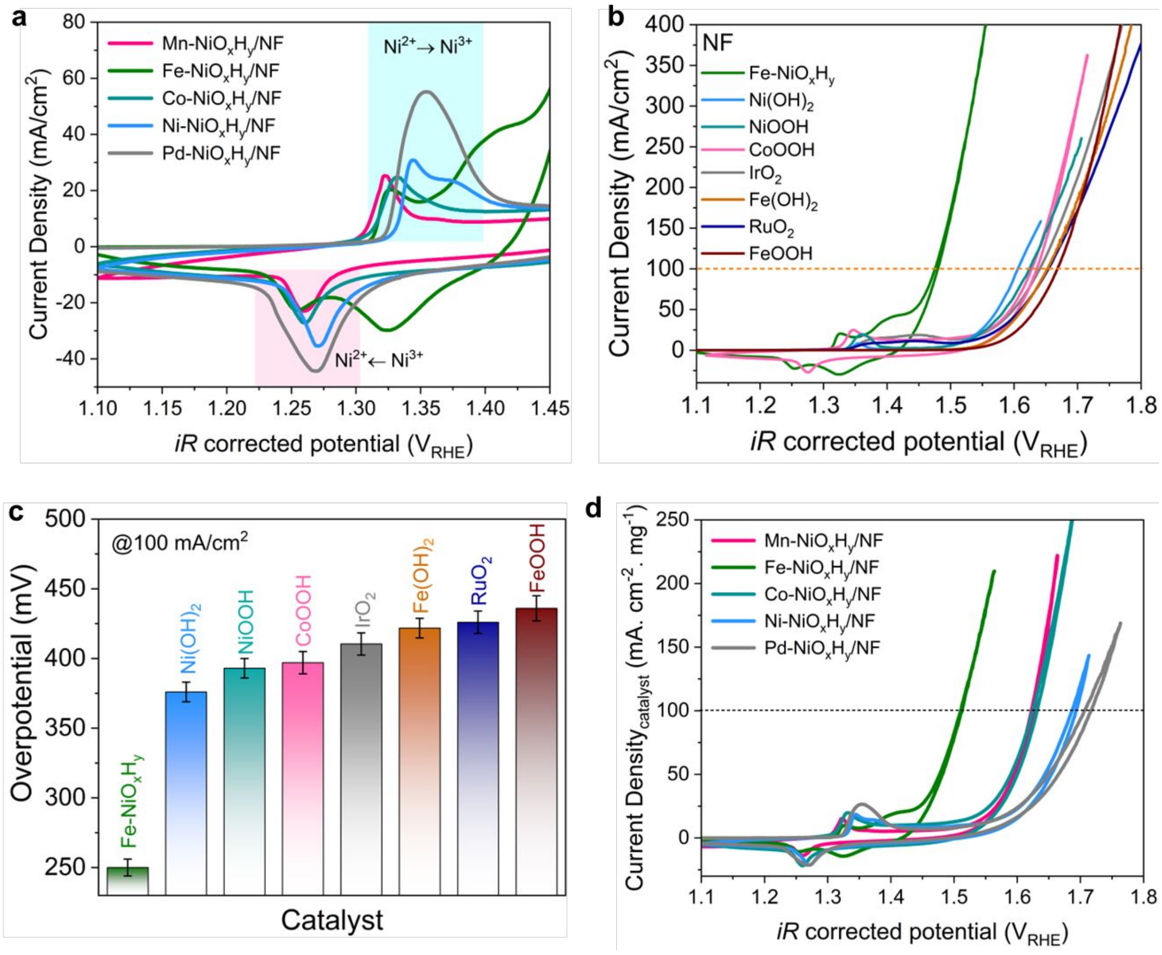

Figure S6. (a) Comparison of cyclic voltammograms (CVs) highlighting the metal redox transitions in the pre-OER region for the prepared catalysts, recorded at a scan rate of 5 mV s^{-1} in 1.0 M KOH. The zoomed region emphasizes the Ni^{2+}/Ni^{3+} redox transformation and the influence of secondary metal incorporation (Mn, Fe, Co, Ni, and Pd) on the redox behavior. (b) Comparison of CV curves of Fe-NiO_xH_y with other non-noble-metal and noble-metal catalysts supported on nickel foam (NF) under identical conditions in 1 M KOH. (c) The comparison of the OER overpotentials (with error bars) at 100 mA. cm^{-2}. The results highlight the superior electrochemical performance of the Fe-NiO_xH_y catalyst compared to the other state-of-the-art catalysts, demonstrating its enhanced catalytic activity and potential for efficient applications. (d) Comparison of CV normalized by the mass of the catalyst grown on NF recorded at a scan rate of 5 mV s^{-1} in 1.0 M KOH. The cyclic voltammograms in the pre-OER region (~1.25-1.40 V vs. RHE) display pronounced redox transitions corresponding primarily to the reversible Ni^{2+}/Ni^{3+} oxidation of $Ni(OH)_2$ to NiOOH, which is a characteristic feature of Ni-based electrocatalysts in alkaline media.[1, 2] The incorporation of different secondary transition metals

(Mn, Fe, Co, Ni, and Pd) into the NiO_xH_y lattice leads to noticeable variations in the peak positions and intensities, indicating modulation of the electronic structure and local coordination environment of the active Ni centers.[2] Specifically, the anodic peaks observed around ~1.32 V (Mn-NiO_xH_y/NF), ~1.33 V (Co-NiO_xH_y/NF), ~1.34 V (Ni-NiO_xH_y/NF), ~1.35 V (Pd-NiO_xH_y/NF), and ~1.38 V (Fe-NiO_xH_y /NF) vs. RHE are mainly attributed to the Ni^{2+} → Ni^{3+} transition, while the corresponding cathodic peaks arise from the reverse Ni^{3+} → Ni^{2+} reduction. The slight shifts among the catalysts originate from electronic interactions between Ni sites and the incorporated metals, which alter the oxidation potential of Ni through inductive effects and changes in d-band occupancy.[2, 3] In addition, partial redox contributions from the secondary metals, such as Co^{2+}/Co^{3+}, Mn^{2+}/Mn^{3+}, and possible Fe^{3+}/Fe^{4+} transitions, may overlap with the Ni redox process, further influencing the peak shape and position. Such electronic coupling between Ni and the secondary metal species is widely reported to modify the energetics of NiOOH formation and is closely associated with the catalytic activation of Ni-based OER electrocatalysts in alkaline electrolytes.[1, 4-6]

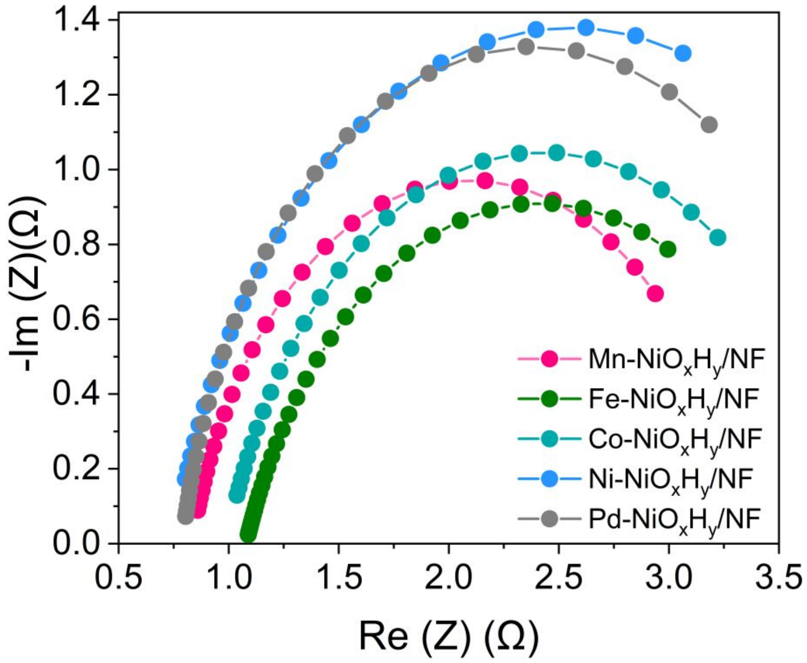

Figure S7. Nyquist plots obtained from electrochemical impedance spectroscopy (EIS) of different self-supported M-NiO_xH_y/NF electrodes (M = Mn, Fe, Co, Ni, Pd) in 1 M KOH measured at 1.538 V_{RHE}.

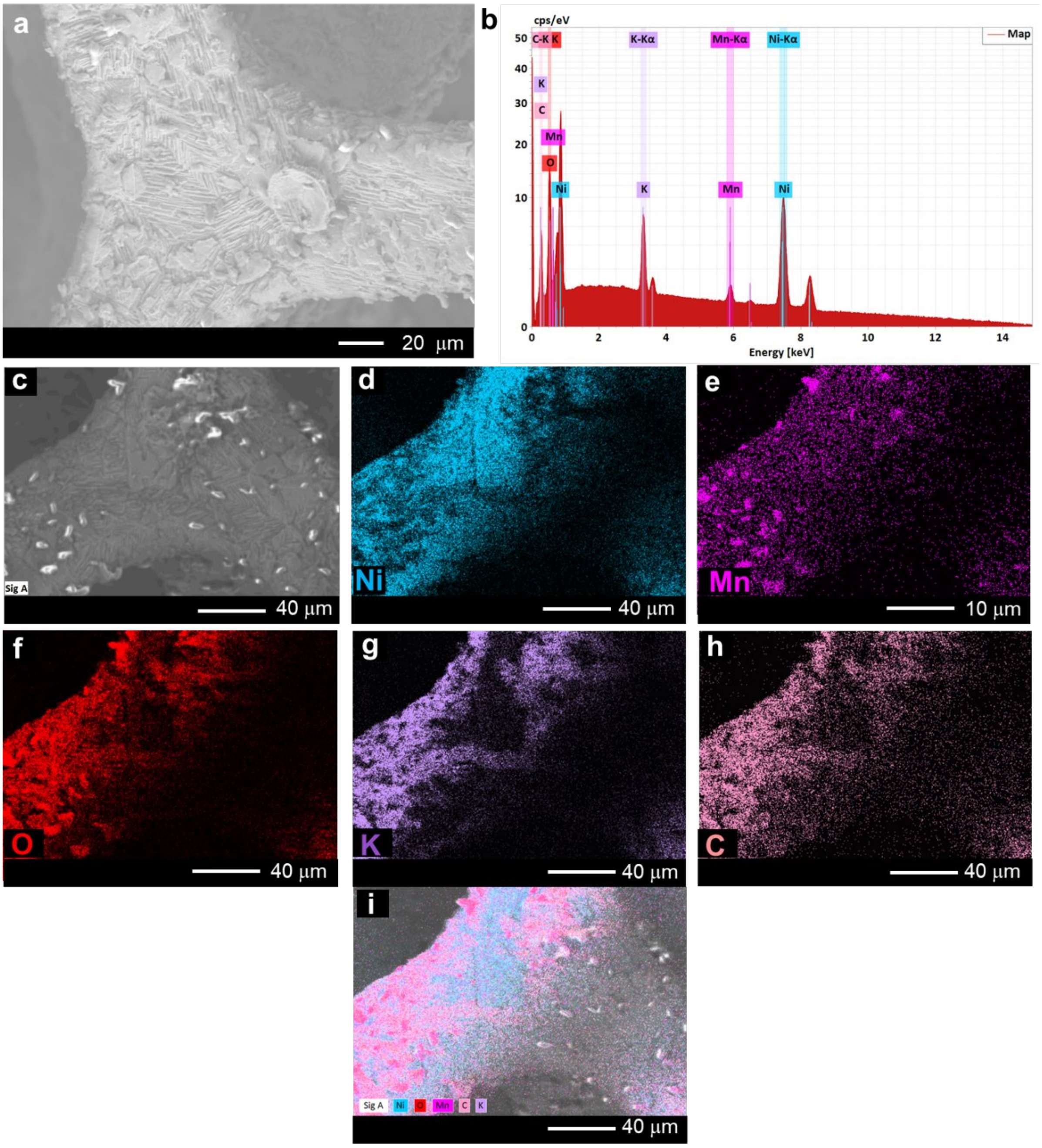

Figure S8. (a) SEM images of the Mn-NiO_xH_y/NF self-supported electrode after electrochemical reconstruction under OER conditions in 1 M KOH and corresponding (b) EDX spectra confirming the presence of different elements in the sample. (c-i) The SEM-EDX elemental mapping for (d) Ni, (e) Mn, (f) O, (g) K, (h) C and (i) combined elemental overlay, demonstrating elemental distribution of the constituent elements across the catalyst surface.

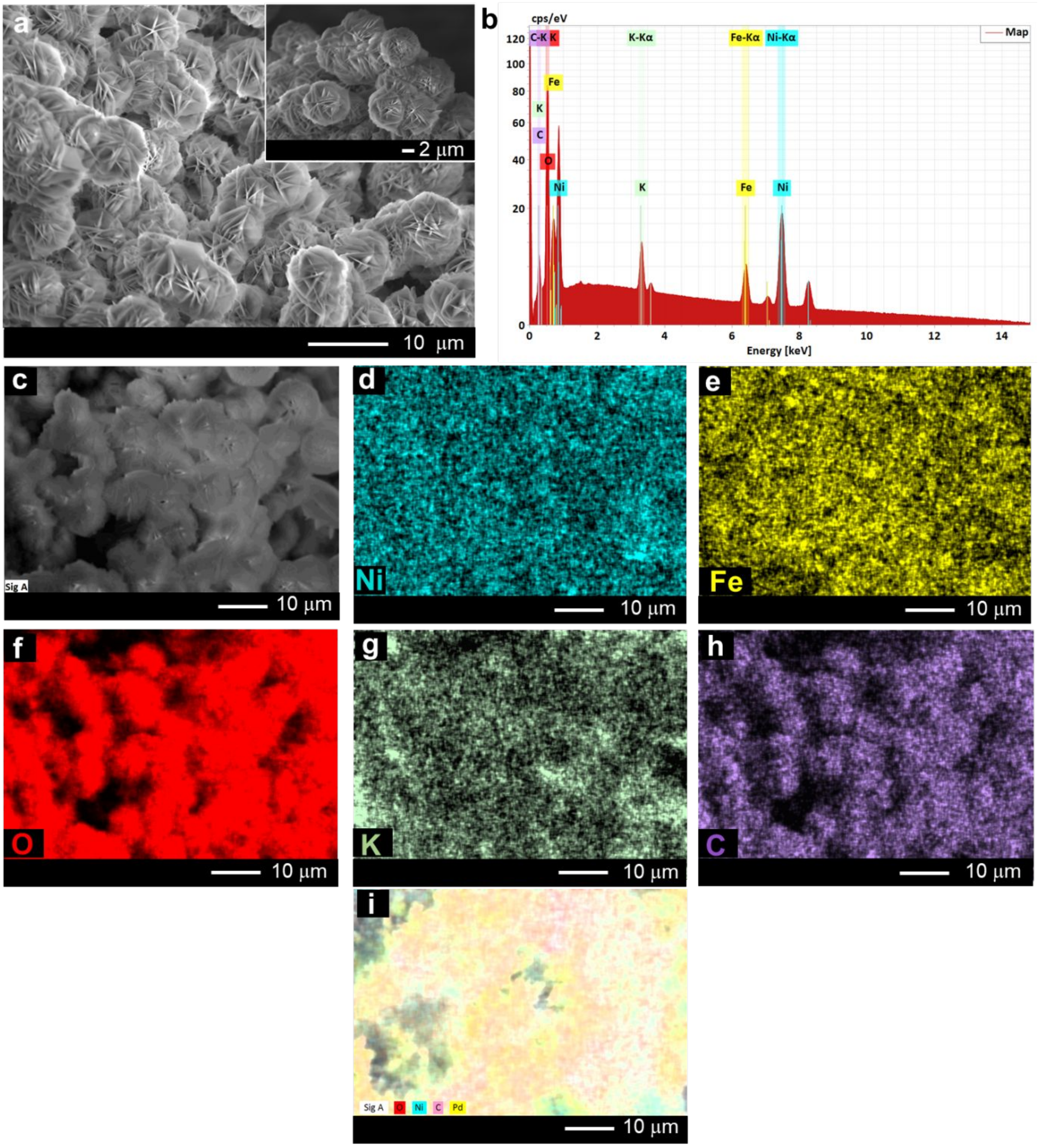

Figure S9. (a) SEM images of the Fe-NiO_xH_y/NF self-supported electrode after electrochemical reconstruction under OER conditions in 1 M KOH and corresponding (b) EDX spectra confirming the presence of different elements in the sample. (c-i) The SEM-EDX elemental mapping for (d) Ni, (e) Fe, (f) O, (g) K, (h) C and (i) combined elemental overlay, demonstrating elemental distribution of the constituent elements across the catalyst surface.

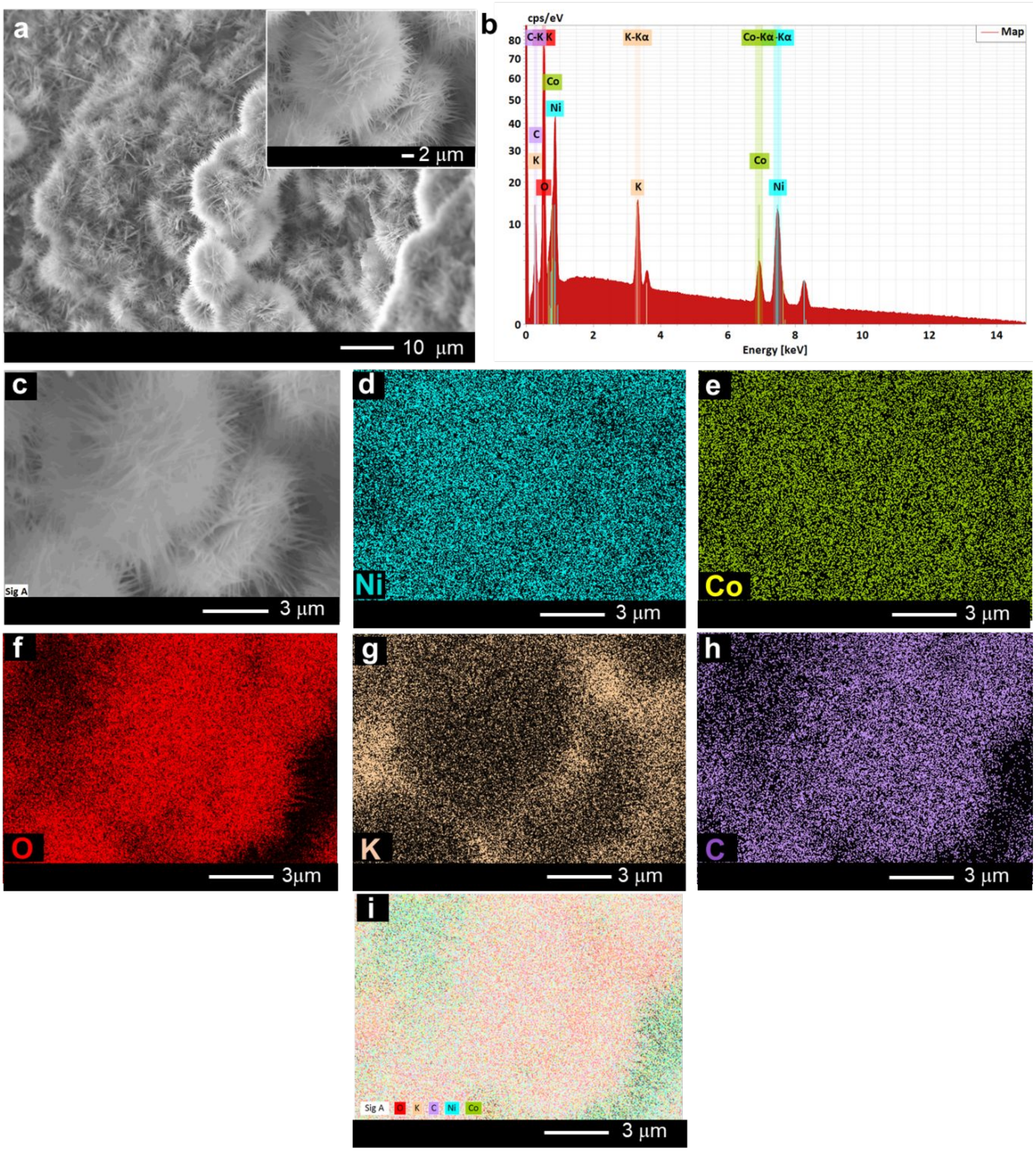

Figure S10. (a) SEM images of the Co-NiO_xH_y/NF self-supported electrode after electrochemical reconstruction under OER conditions in 1 M KOH and corresponding (b) EDX spectra confirming the presence of different elements in the sample. (c-i) The SEM-EDX elemental mapping for (d) Ni, (e) Co, (f) O, (g) K, (h) C and (i) combined elemental overlay, demonstrating elemental distribution of the constituent elements across the catalyst surface.

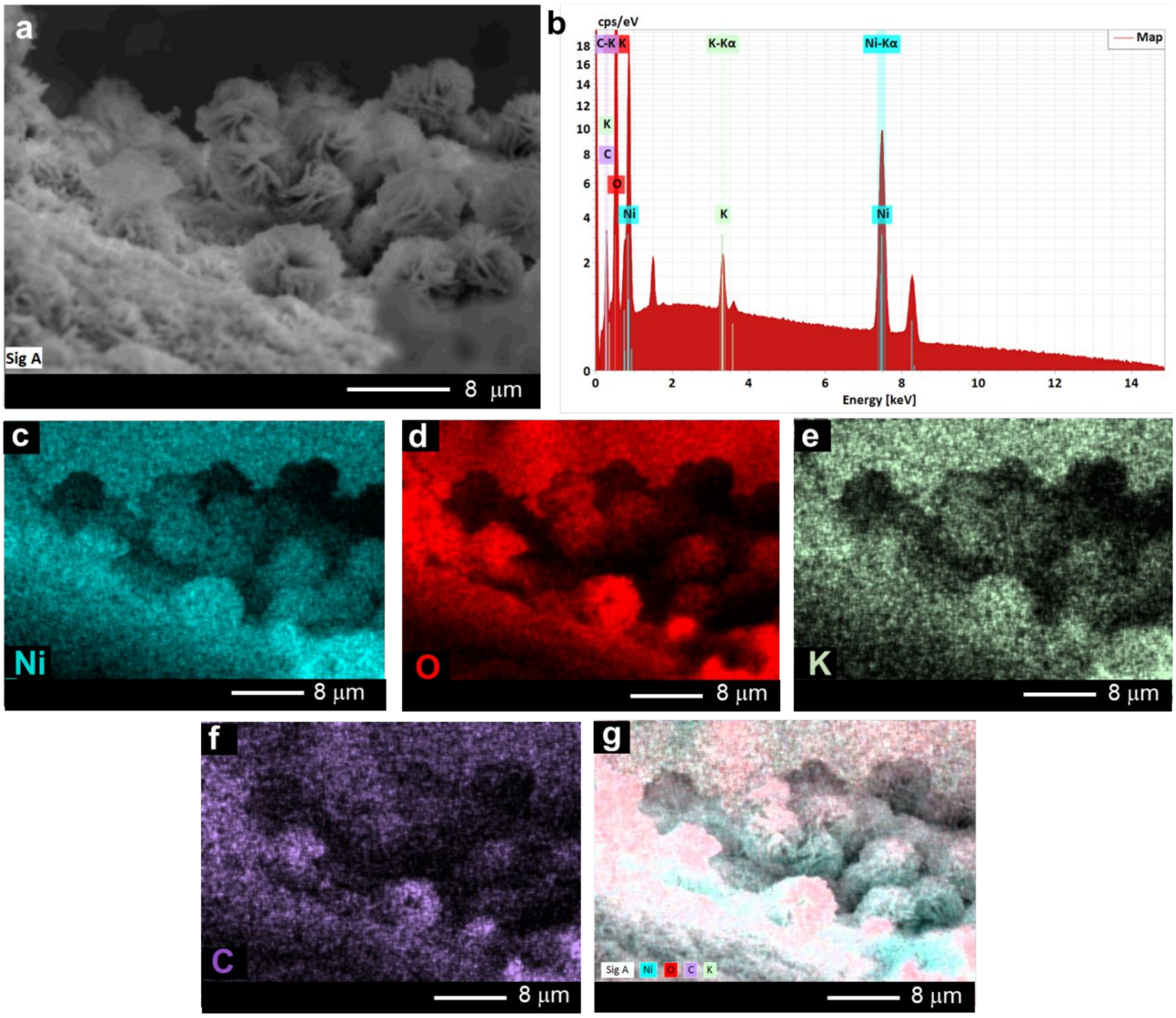

Figure S11. (a) SEM images of the Ni-NiO_xH_y/NF self-supported electrode after electrochemical reconstruction under OER conditions in 1 M KOH and corresponding (b) EDX spectra confirming the presence of different elements in the sample. (c-g) The SEM-EDX elemental mapping for (c) Ni, (d) O, (e) K, (f) C and (g) combined elemental overlay, demonstrating elemental distribution of the constituent elements across the catalyst surface.

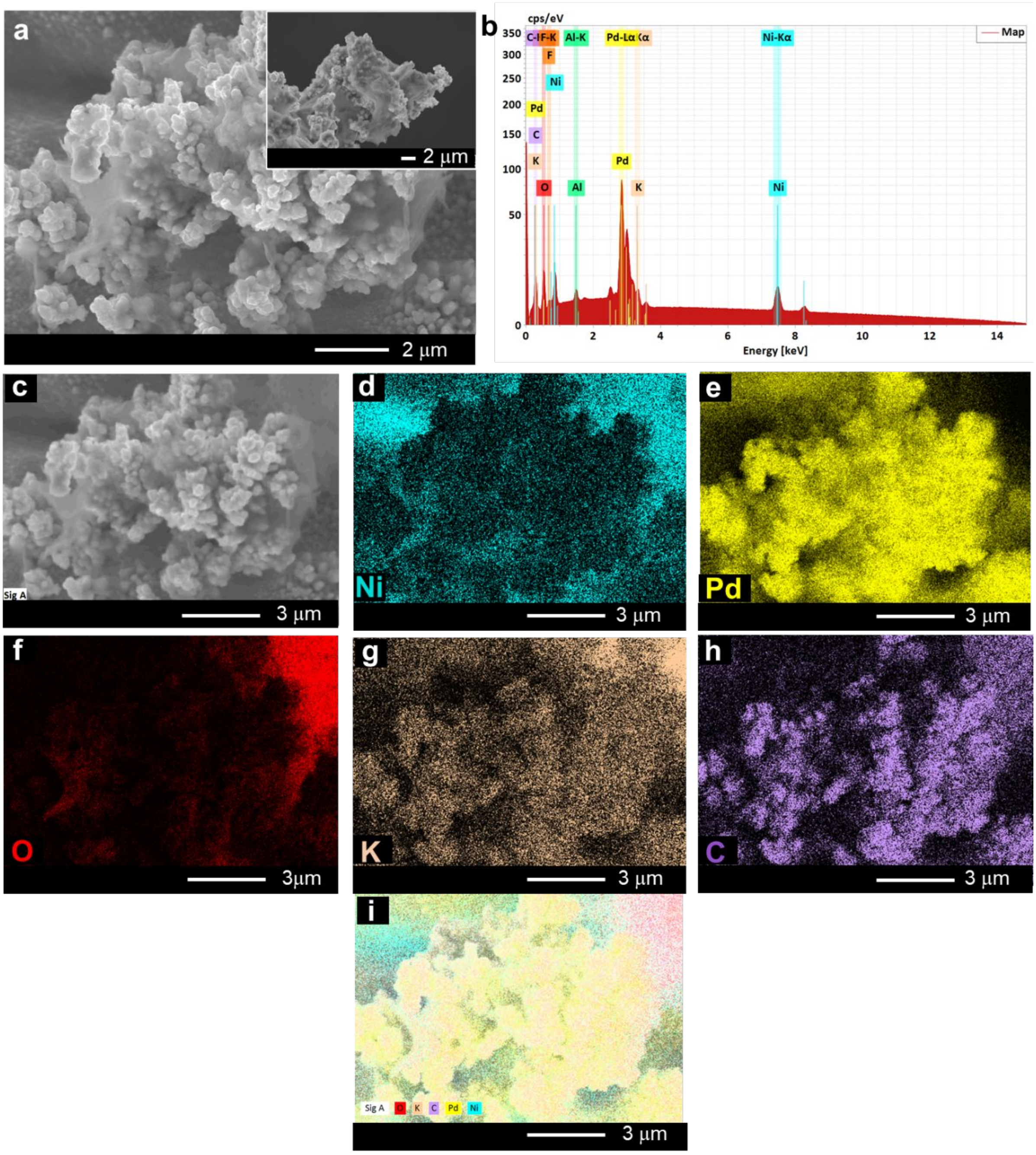

Figure S12. (a) SEM images of the Pd-NiO_xH_y/NF self-supported electrode after electrochemical reconstruction under OER conditions in 1 M KOH and corresponding (b) EDX spectra confirming the presence of different elements in the sample. (c-i) The SEM-EDX elemental mapping for (d) Ni, (e) Pd, (f) O, (g) K, (h) C and (i) combined elemental overlay, demonstrating elemental distribution of the constituent elements across the catalyst surface.

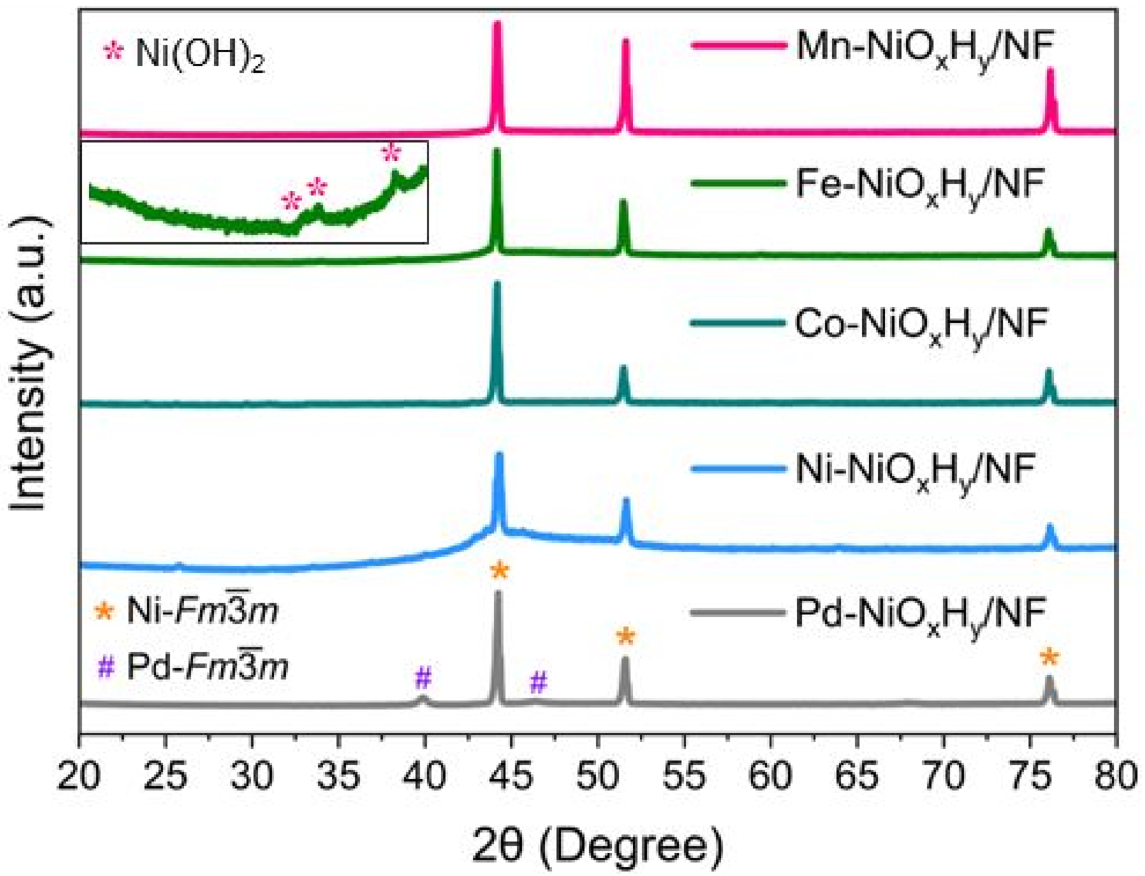

Figure S13. Powder XRD of the self-supported M-NiO_xH_y/NF (M= Mn, Fe, Co, Ni, Pd) catalyst grown on nickel foam after the electrochemical reconstruction under OER conditions in 1 M KOH. The powder XRD patterns primarily display reflections corresponding to the NiO_xH_y phase grown on the Ni foam substrate, while distinct peaks associated with Mn-, Fe-, or Co-containing hydroxide phases are not clearly resolved. This can be attributed to the ultrathin nanosheet morphology, low dopant concentration, and the similar ionic radii of the secondary metal ions to Ni^{2+}, resulting in minimal lattice distortion detectable by powder XRD. Electrochemical reconstruction during catalytic operation leads to increased structural disorder and amorphization of the hydroxide framework, which further broadens or suppresses diffraction features.

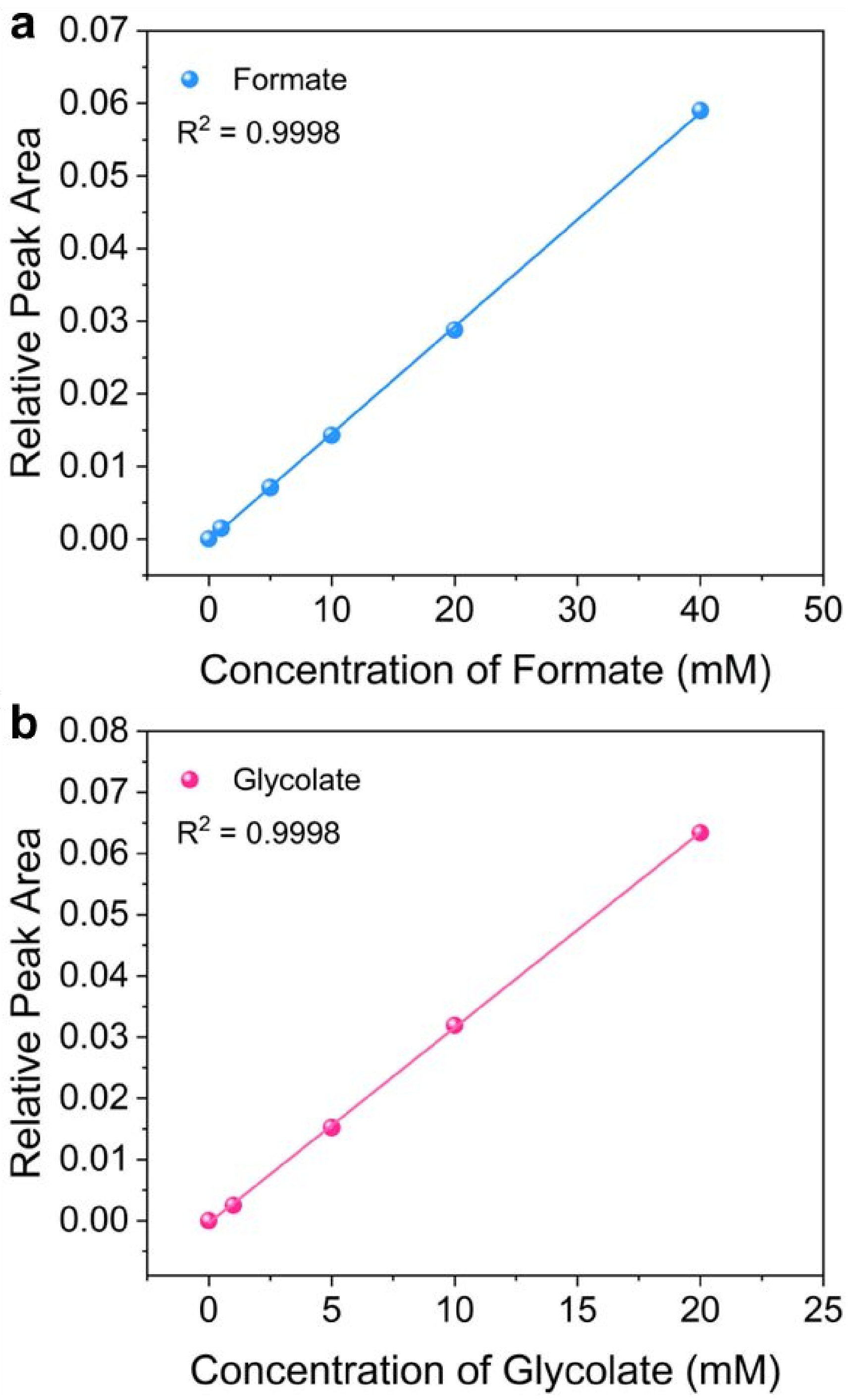

Figure S14. ^{1}H NMR calibration curves for (a) formate and (b) glycolate. Standard solutions of known concentrations were prepared in 1.0 M KOH and analyzed under identical ^{1}H NMR acquisition conditions. The calibration plots were obtained by correlating the ratio of the integrated proton signal of formate or glycolate to that of the internal standard Dimethyl sulfoxide (DMSO) with the corresponding analyte concentration. The resulting linear relationships were employed to determine product concentrations in electrolysis samples acquired under the same conditions.

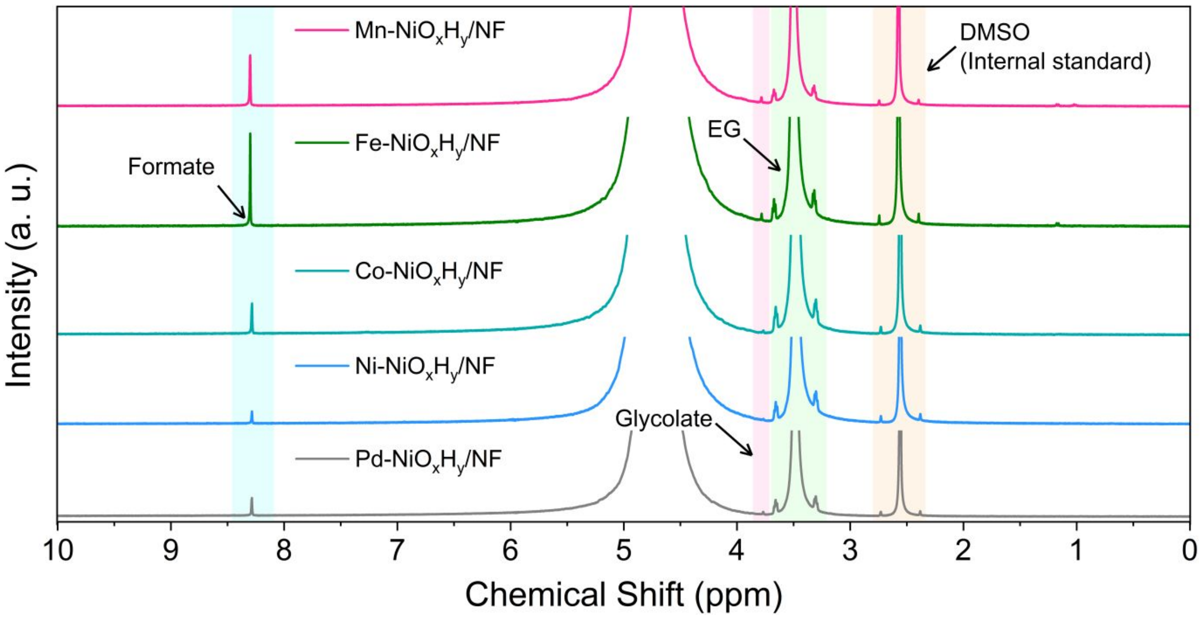

Figure S15. ^{1}H NMR spectra of post-electrolysis electrolytes collected after 60 min of EGOR in 1.0 M EG dissolved in 1.0 M KOH at an applied potential of 1.38 V vs RHE. Electrolysis was carried out using preactivated, self-supported M-NiO_xH_y/NF electrodes (M = Mn, Fe, Co, Ni, Pd). Catalyst pre-activation was achieved via CV under OER conditions (potential window: 1.10-1.52 V vs RHE; scan rate: 20 mV s^{-1}) for 50 cycles in 1.0 M KOH, followed by EGOR performed at 1.38 V vs RHE for 60 min in 1.0 M EG dissolved in 1.0 M KOH. DMSO was used as the internal standard for quantitative analysis.

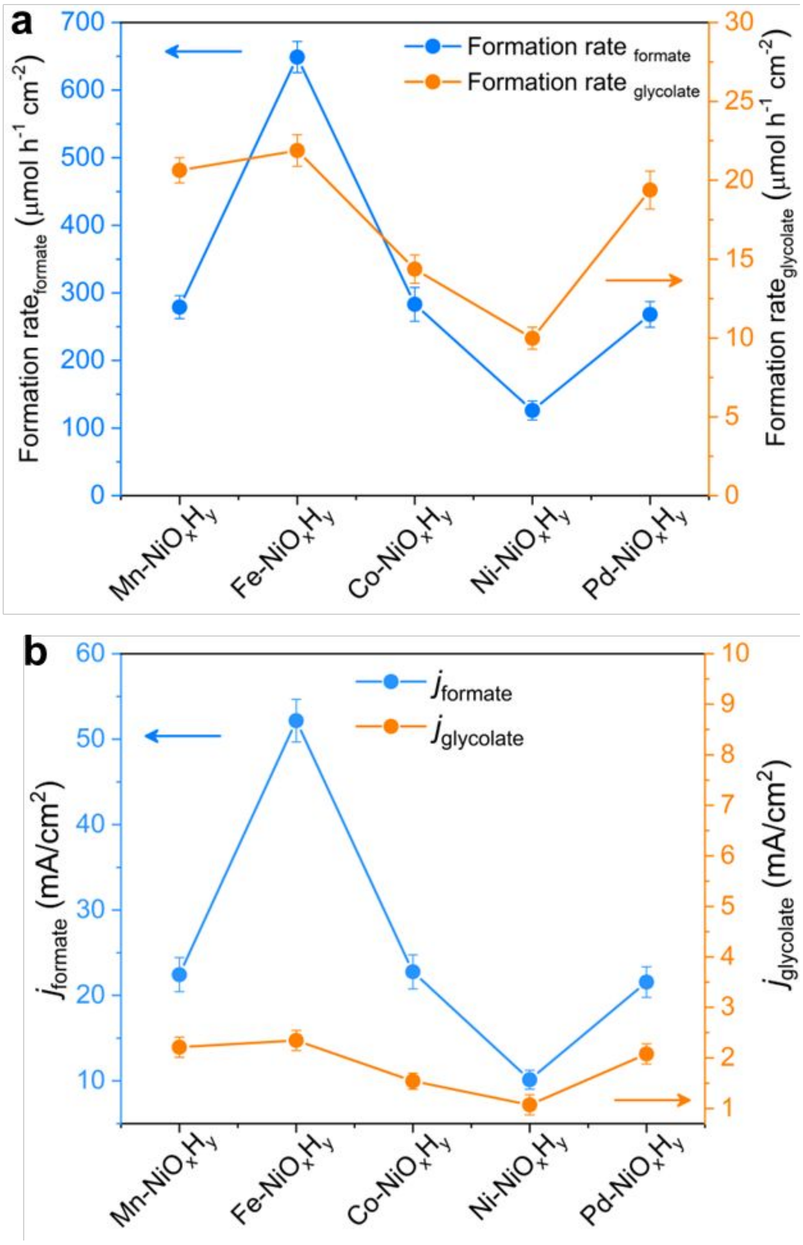

Figure S16. Comparative evaluation of EGOR performance on pre-activated, self-supported M-NiO_xH_y/NF electrodes (M = Mn, Fe, Co, Ni, Pd). Panels (a) and (b) present the product formation rates and partial current densities, respectively, for formate and glycolate obtained during bulk electrolysis conducted at 1.38 V vs RHE for 60 min in 1.0 M EG dissolved in 1.0 M KOH. Catalyst pre-activation was performed by CV under OER conditions (potential window: 1.10-1.52 V vs RHE; scan rate: 20 mV s^{-1}) for 50 cycles in 1.0 M KOH, followed by EGOR.

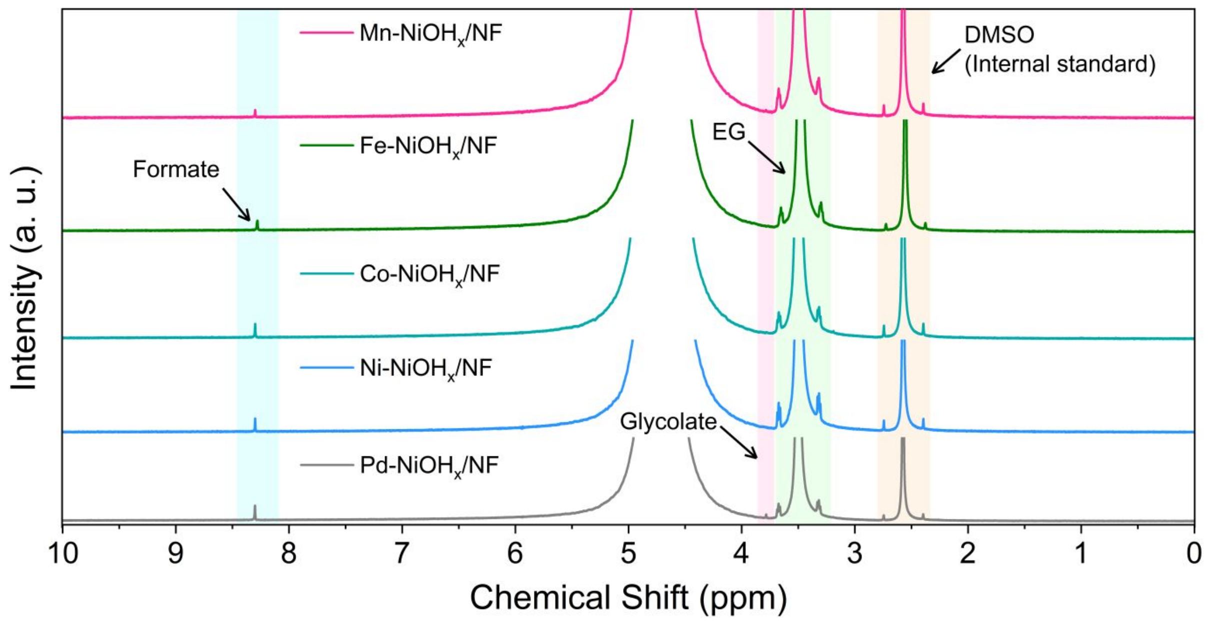

Figure S17. ^{1}H NMR spectra of the post-electrolysis electrolyte collected after 60 min of ethylene glycol (EG) electrooxidation in 1.0 M EG dissolved in 1.0 M KOH at an applied potential of 1.38 V vs. RHE. Electrolysis was performed using self-supported M-NiO$_x$H$_y$/NF (M = Mn, Fe, Co, Ni, Pd) catalysts grown directly on NF electrodes. DMSO was employed as the internal standard for quantitative analysis.

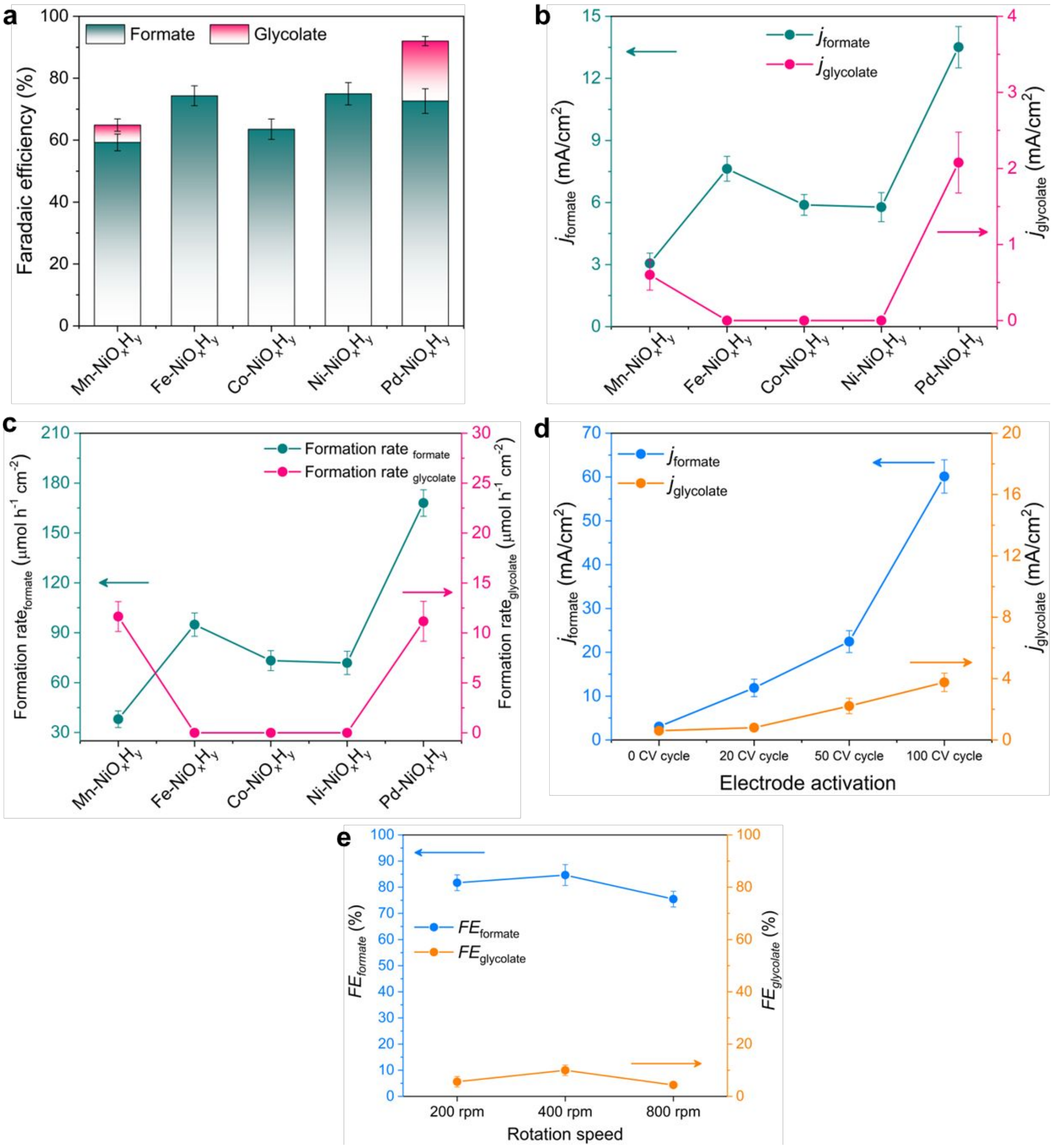

Figure S18. Comparative evaluation of EGOR performance on as-prepared, self-supported M-NiO_xH_y/NF electrodes (M=Mn, Fe, Co, Ni, Pd). Panels (a-c) present the (a) Faradaic efficiency (FE), (b) partial current density, and (c) product formation rate for formate and glycolate obtained during bulk electrolysis conducted at 1.38 V vs RHE in 1.0 M EG dissolved in 1.0 M KOH. Panel (d) illustrates the effect of electrochemical pre-activation on product selectivity, showing the partial current densities of formate and glycolate measured on the Mn-NiO_xH_y/NF electrode after varying numbers of cyclic voltammetry (CV) activation cycles performed under oxygen evolution reaction (OER) conditions (potential window: 1.10-1.52 V vs RHE; scan rate: 20 mV s^{-1}) in 1.0 M KOH, followed by EGOR at 1.38 V vs RHE in 1.0 M EG dissolved in 1.0 M KOH. (e) Effect of stirring rate on product selectivity during EGOR on Mn-

NiO_xH_y/NF. Faradaic efficiency of formate and glycolate are shown at 200, 400, and 800 rpm under EGOR conditions (1.38 V vs RHE in 1.0 M EG + 1.0 M KOH).

The potential difference between EGOR and OER for Fe-NiO_xH_y/NF arises from the electronic modulation of Ni active sites by Fe incorporation. The formation of Ni-O-Fe motifs promotes the Ni^{2+}/Ni^{3+} redox transition and stabilizes high-valent NiOOH species, which serve as the active phase for both OER and ethylene glycol oxidation in alkaline media. Consequently, the activation barriers for hydroxide adsorption and reaction intermediates are reduced for both processes.[1-3] We investigated the role of mass transport in ethylene glycol electrooxidation and found that stirring of the magnetic bead critically influences product selectivity. An optimum stirring rate of 400 rpm ensures sufficient mass transport while avoiding mechanical loss of the catalyst, whereas lower or higher rates lead to mass-transport limitations or catalyst detachment, respectively.

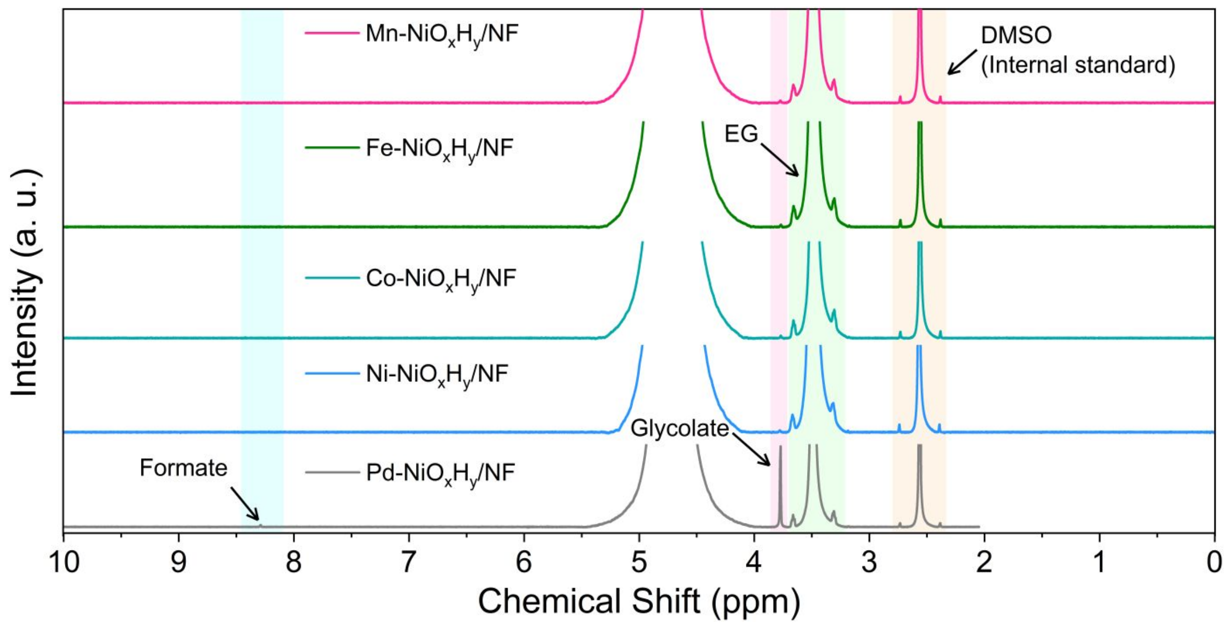

Figure S19. ^{1}H NMR spectra of the post-electrolysis electrolyte collected after 60 min of ethylene glycol (EG) electrooxidation in 1.0 M EG dissolved in 1.0 M KOH at an applied potential of 0.843 V vs. RHE (low potential EGOR). Electrolysis was performed using as-prepared self-supported M-NiO_xH_y/NF (M = Mn, Fe, Co, Ni, Pd) catalysts grown directly on NF electrodes. DMSO was employed as the internal standard for quantitative analysis.

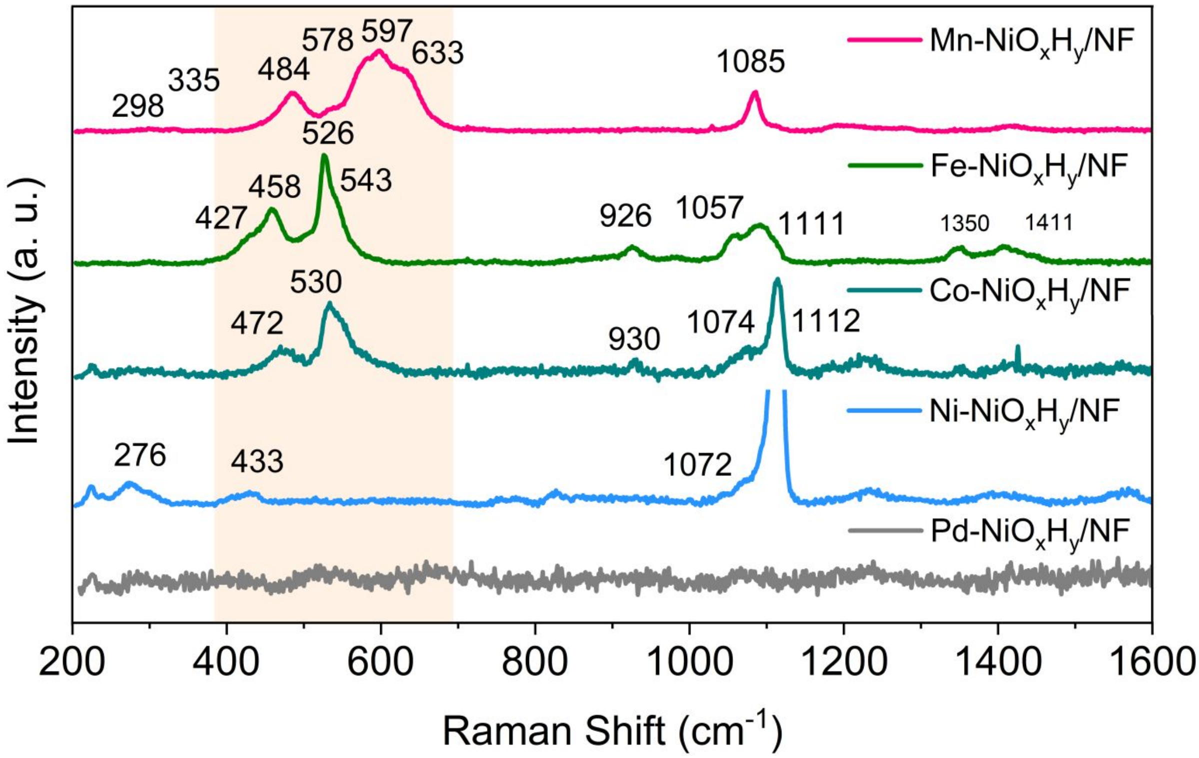

Figure S20. Ex-situ Raman spectra obtained, using the 458 nm wavelength of an Ar^+ laser for excitation on OER-reconstructed electrodes for all M-NiO_xH_y/NF catalysts (M = Mn, Fe, Co, Ni and Pd) in 1.0 M KOH, illustrating metal-oxygen vibrational modes and structural modifications induced by electrochemical activation.[7-9] The Raman bands observed in **Figure 2a** the 400-550 cm^{-1} region correspond to the Ni^{2+}-O lattice vibrations characteristic of $Ni(OH)_2$-based hydroxide structures. Notably, the Ni-O stretching mode appears at a slightly lower wavenumber for the Ni and Fe-incorporated samples compared with the Co- and Mn-modified counterparts, indicating a reduced Ni-O bond force constant caused by lattice distortion and the formation of Ni-O-Fe interactions within the hydroxide framework. Similar dopant-induced modulation of Ni-O vibrational modes in Ni-based hydroxides and layered double hydroxides has been reported previously.[3, 10, 11]

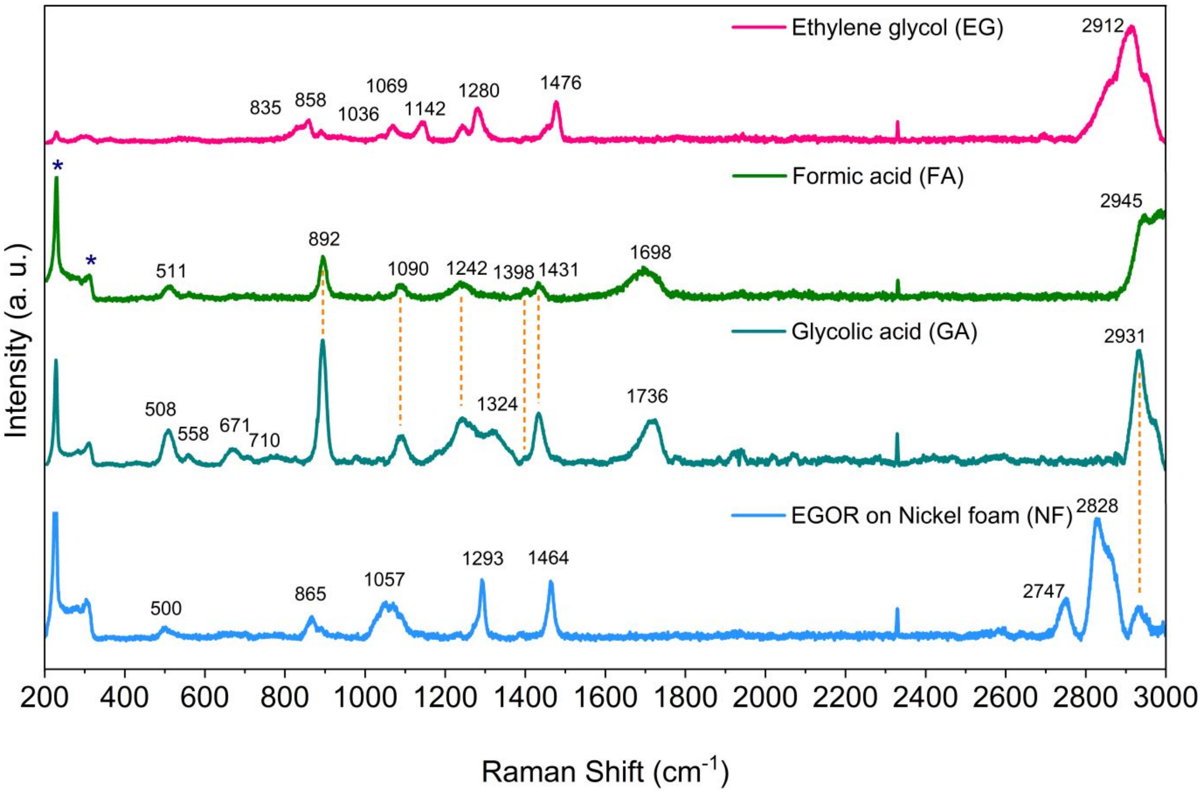

Figure S21. Reference and quasi in-situ Raman spectra relevant to EGOR obtained with a 458 nm wavelength of an Ar^+ laser. Raman spectra of reference compounds (substrate and potential products), ethylene glycol (EG), formic acid (FA), and glycolic acid (GA) were recorded for a spectral assignment. The quasi in-situ Raman spectra recorded on bare nickel foam during EGOR at an applied potential of 1.38 V vs RHE was taken after 60 min of electrolysis in 1 M EG + 1 M KOH. The herein displayed spectral marker bands can be used to assign the key vibrational features associated with (bound) substrates, reaction intermediates and products formed during EGOR.[12-16]

Table S1. Atomic percentages of metals in nickel-based catalysts (M-NiO_xH_y/NF, M = Mn, Fe, Co, Ni, Pd) determined by SEM-EDX for the as-prepared samples and after 50 CV cycles of OER activation (potential window: 1.10-1.52 V vs RHE; scan rate: 20 mV s^{-1}) in 1.0 M KOH.

**Sample**	**Atomic % (EDX)**							
	**Mn**	**Fe**	**Co**	**Ni**	**Pd**	**O**	**C**	
Mn-NiO_xH_y/NF	3.47			28.25		47.05	18.57	
Fe-NiO_xH_y/NF		2.56		32.18		54.19	11.07	
Co-NiO_xH_y/NF			6.61	27.53		51.20	14.66	
Ni-NiO_xH_y/NF				21.08		57.80	21.12	
Pd-NiO_xH_y/NF				11.12	38.64	50.24	0.00	
	**Atomic % (EDX) after OER Activation**							
	Mn	Fe	Co	Ni	Pd	O	C	K
Mn-NiO_xH_y/NF	1.03			31.45		32.59	29.69	5.23
Fe-NiO_xH_y/NF		3.24		23.19		58.27	13.01	2.28
Co-NiO_xH_y/NF			3.49	16.03		58.87	18.00	3.61
Ni-NiO_xH_y/NF				31.09		49.02	18.47	1.42
Pd-NiO_xH_y/NF				13.05	44.15	41.44	0.0	1.36

Table S2. Comparison of the OER overpotential (η) and other crucial electrochemical parameters of the self-supported catalysts developed in this study with those of well-established OER catalysts reported in the literature.

Catalysts	*Substrate*	*Over potential (η_{100})*	*Current density (mA/cm^2)*	*Tafel Slope (mV/dec)*	*Electrolyte*	*Ref.*
$NiFeCr_{0.1}$/NF	NF	268	100	29.3	1M KOH	17
NiCoFe/NF (trimetallic)	NF	293	100	63	1M KOH	18
CoSn-Ni_3S_2@NF	NF	321	200	69	1M KOH	19
Hierarchical NiFe LDH/N-doped Co/NF	NF	262	100	31.4	1M KOH	20
ZIF-67 derived NiFeCo-P/NF	NF	278	100	25.24	1M KOH	21
S-(Ni,Fe)OOH	NF	281	100	48.9	1M KOH	22
Ni_2P-Fe_2P	NF	305	100	59	1M KOH	23
NiMoN@NiFeN	NF	277	100	58.6	1M KOH	24
B-Co_2Fe LDH	NF	246	100	39.2	1M KOH	25
NiVIr LDH	NF	272	100	38	1M KOH	26
Ru-MnFeP	NF	282	100	69	1M KOH	27
Fe-doped β-$Ni(OH)_2$	NF	275	100	53	1M KOH	28
NiFe LDH@NiCoP	NF	258	100	48.6	1M KOH	29
NiFeRu LDH	NF	258	100	32.4	1M KOH	30
Ni-ZIF/Ni-B	NF	320	100	57	1M KOH	31
FeP_2	NF	315	100	56	1M KOH	32

Cu@CoFe LDH	NF	300	100	44.4	1M KOH	[33]
**Fe-NiO_xH_y**	**NF**	**250±7**	**100**	**35±2**	**1M KOH**	**This work**

Table S3. Comparison of the EGOR performance and other crucial electrochemical parameters of the self-supported catalysts developed in this study with those of well-established EGOR catalysts for formate selectivity reported in the literature.

Catalysts	*Substrate*	*Electrolyte Composition*	*Current density (mA/cm^2)*	*Faradaic efficiency to formate (%)*	*Ref*
NiS	NF	1.0 M KOH + 1.0 M EG	50	>80	34
$NiSe_2$	NF	1.0 M KOH + 1.0 M EG	50	>80	34
Ni@NF	NF	1.0 M KOH + 0.1 M EG	27.2	~50	35
Ni_xS	NF	1.0 M KOH + 0.5 M EG	50	~78	36
CoP	NF	1.0 M KOH + 1.0 M EG	100	~68	36
$Ni(OH)_2/Ni$	NF	10 M KOH with 0.3 M EG	100	~90	37
NiOOH/NF	NF	1.0 M KOH + 0.1 M EG	100	88	38
CoFe-LDH	NF	1.0 M KOH + 0.1 M EG	10	49.7	39
3D NiCo-O nanowires	NF	1.0 M KOH + 0.1 M EG	10	92	40
Ni_3S_2@NiFeMn-LDH	NF	1.0 M KOH + 1.0 M EG	100	90	41
**Fe-NiO_xH_y**	NF	1.0 M KOH + 1.0 M EG	52 ± 2.5	>90	**This work**

Table S4. Comparison of the EGOR performance and other crucial electrochemical parameters of the self-supported catalysts developed in this study with those of well-established EGOR catalysts for glycolate selectivity reported in the literature.

Catalysts	***Substrate***	***Electrolyte Composition***	***Current density (mA/cm^2)***	***Faradaic efficiency to glycolate (%)***	***Ref***
Pt-Au bimetallic nanoparticles	Carbon support	1.0 M NaOH + 0.1 M EG	-	>50	42
PdAg nanoparticles	NF	1.0 M KOH + 1.0 M EG	-	>90	43
Au electrode	Au disk	1.0 M NaOH + 1.0 M EG	-	>95	44
Hollow PtAg nanowires	Carbon paper	1.0 M KOH + 1.5 M EG	355	~97	45
Pt electrode	Pt disk	1.0 M NaOH + 1.0 M EG	-	>85	46
PdAg alloy	NF	1.0 M KOH + 0.1 M EG	-	>80	47
Pd@Bi-PdBi heterodime	NF	1.0 M KOH + 1.0 M EG	-	>90	48
Ir_1Pd	NF	1.0 M PBS + 1.0 M EG	56.2	76.3	49
$Pt-Ni(OH)_2$	Carbon electrode	1.0 M KOH + 1.0 M EG	250	>90	50
$Pd/NiMoO_4$	NF	1.0 M NaOH + 1.0 M EG	-	>90	51
**$Pd-NiO_xH_y$**	NF	1.0 M KOH + 1.0 M EG	200	92.5	**This work**

References

1. Trotochaud, L.; Young, S. L.; Ranney, J. K.; Boettcher, S. W., Nickel–Iron Oxyhydroxide Oxygen-Evolution Electrocatalysts: The Role of Intentional and Incidental Iron Incorporation. *J. Am. Chem. Soc.* **2014,** *136*, 6744-6753.
2. Corrigan, D. A., The Catalysis of the Oxygen Evolution Reaction by Iron Impurities in Thin Film Nickel Oxide Electrodes. *Journal of The Electrochemical Society* **1987,** *134*, 377.
3. Friebel, D.; Louie, M. W.; Bajdich, M.; Sanwald, K. E.; Cai, Y.; Wise, A. M.; Cheng, M.-J.; Sokaras, D.; Weng, T.-C.; Alonso-Mori, R.; Davis, R. C.; Bargar, J. R.; Nørskov, J. K.; Nilsson, A.; Bell, A. T., Identification of Highly Active Fe Sites in (Ni,Fe)OOH for Electrocatalytic Water Splitting. *J. Am. Chem. Soc.* **2015,** *137*, 1305-1313.
4. Xing, C.; Musharavati, F.; Li, H.; Zalezhad, E.; Hui, O. K. S.; Bae, S.; Cho, B.-Y., Synthesis, characterization, and properties of nickel–cobalt layered double hydroxide nanostructures. *RSC Advances* **2017,** *7*, 38945-38950.
5. Chang, H.-W.; Lee, C.-H.; Yang, S.-H.; Chiu, K.-C.; Liu, T.-Y.; Tsai, Y.-C., Nickel–Cobalt Layered Double Hydroxide Nanosheet-Decorated 3D Interconnected Porous Ni/SiC Skeleton for Supercapacitor. **2024,** *29*, 5664.
6. Paulraj, A. R.; Kiros, Y., La0.1Ca0.9MnO3/Co3O4 for oxygen reduction and evolution reactions (ORER) in alkaline electrolyte. *Journal of Solid State Electrochemistry* **2018,** *22*, 1697-1710.
7. Yang, H.; Vijaykumar, G.; Chen, Z.; Hausmann, J. N.; Mondal, I.; Ghosh, S.; Nicolaus, V. C. J.; Laun, K.; Zebger, I.; Driess, M.; Menezes, P. W., In Situ Reconstruction of Helical Iron Borophosphate Precatalyst toward Durable Industrial Alkaline Water Electrolysis and Selective Oxidation of Alcohols. *Adv. Funct. Mater.* **2023,** *33*, 2303702.
8. Ghosh, S.; Dasgupta, B.; Kalra, S.; Ashton, M. L. P.; Yang, R.; Kueppers, C. J.; Gok, S.; Alonso, E. G.; Schmidt, J.; Laun, K.; Zebger, I.; Walter, C.; Driess, M.; Menezes, P. W., Evolution of Carbonate-Intercalated γ-NiOOH from a Molecularly Derived Nickel Sulfide (Pre)Catalyst for Efficient Water and Selective Organic Oxidation. *Small* **2023,** *19*, 2206679.
9. Laan, P. C. M.; de Zwart, F. J.; Wilson, E. M.; Troglia, A.; Lugier, O. C. M.; Geels, N. J.; Bliem, R.; Reek, J. N. H.; de Bruin, B.; Rothenberg, G.; Yan, N., Understanding the Oxidative Properties of Nickel Oxyhydroxide in Alcohol Oxidation Reactions. *ACS Catal.* **2023,** *13*, 8467-8476.
10. Louie, M. W.; Bell, A. T., An Investigation of Thin-Film Ni–Fe Oxide Catalysts for the Electrochemical Evolution of Oxygen. *J. Am. Chem. Soc.* **2013,** *135*, 12329-12337.
11. Gong, M.; Li, Y.; Wang, H.; Liang, Y.; Wu, J. Z.; Zhou, J.; Wang, J.; Regier, T.; Wei, F.; Dai, H., An Advanced Ni–Fe Layered Double Hydroxide Electrocatalyst for Water Oxidation. *J. Am. Chem. Soc.* **2013,** *135*, 8452-8455.
12. Maxwell, D. S.; Kendrick, I.; Mukerjee, S., Operando Raman Spectroscopy Reveals Degradation Byproducts from Ionomer Oxidation in Anion Exchange Membrane Water Electrolyzers. *J. Am. Chem. Soc.* **2024,** *146*, 22431-22444.
13. Liubimovskii, S. O.; Novikov, V. S.; Ustynyuk, L. Y.; Ivchenko, P. V.; Prokhorov, K. A.; Kuzmin, V. V.; Sagitova, E. A.; Godyaeva, M. M.; Gudkov, S. V.; Darvin, M. E.; Nikolaeva, G. Y., Raman structural study of ethylene glycol and 1,3-propylene glycol aqueous solutions. *Spectrochim Acta A Mol Biomol Spectrosc.* **2023,** *285*, 121927.
14. Leonard, M.; Milosavljevic, B. H. J. P. C. C. P., Spectroscopic and complementary thermodynamic study of liquid, supercooled, and glassy state of ethylene glycol. **2025,** *27*, 23627-23636.
15. Krishnan, K.; Krishnan, R. S., Raman and infrared spectra of ethylene glycol. *Proceedings of the Indian Academy of Sciences - Section A* **1966,** *64*, 111-122.

16. Mohaček-Grošev, V.; Šoštarić, V.; Maksimović, A. J. S. a. P. A., Molecular; spectroscopy, b., Raman spectroscopic evidence of low temperature stability of D,L-glycolic and L-(+)-lactic acid crystals. **2015,** *140*, 35-43.
17. Zhang, Z.; Yang, Z.; Gao, J.; Wu, X.; Yun, J.; Zhang, J., Efficient NiFeCr0.1/NF by incorporating chromium to boost OER catalysis. *Mol. Catal.* **2024,** *557*, 113980.
18. Jung, H. Y.; Park, J. H.; Ro, J. C.; Suh, S. J., Fabrication of Trimetallic Fe–Co–Ni Electrocatalysts for Highly Efficient Oxygen Evolution Reaction. *ACS Omega* **2022,** *7*, 45636-45641.
19. An, W. Y.; Lee, H.; Choi, S. R.; Choi, S.; Cho, H.-S.; Choi, M.; Park, J.-Y., Hierarchically porous Ni foam-supported Co and Sn doped Ni3S2 nanosheets for oxygen evolution reaction electrocatalysts. *J. Mater. Chem. A* **2023,** *11*, 5734-5745.
20. Wang, J.; Wang, Y.; Guo, X.; Chen, M.; Fang, J.; Li, X.; Zhu, W.; Zhuang, Z., Hierarchical NiFe LDH/N-doped Co/nickel foam as highly active oxygen evolution reaction electrode for anion exchange membrane water electrolysis. *Nano Res.* **2025,** *18*, 94907190.
21. Guo, X.; Li, L.; Wang, S.; Zhang, H.; Kuang, Y.; Duan, G.; Cao, B., An in situ formed ZIF-67 derived NiFeCo-P nano-array for accelerating the electrocatalytic oxygen evolution reaction. *Energy adv.* **2024,** *3*, 654-663.
22. Yu, L.; Wu, L.; McElhenny, B.; Song, S.; Luo, D.; Zhang, F.; Yu, Y.; Chen, S.; Ren, Z., Ultrafast room-temperature synthesis of porous S-doped Ni/Fe (oxy)hydroxide electrodes for oxygen evolution catalysis in seawater splitting. *Energy Environ. Sci.* **2020,** *13*, 3439-3446.
23. Wu, L.; Yu, L.; Zhang, F.; McElhenny, B.; Luo, D.; Karim, A.; Chen, S.; Ren, Z., Heterogeneous Bimetallic Phosphide Ni2P-Fe2P as an Efficient Bifunctional Catalyst for Water/Seawater Splitting. *Adv. Funct. Mater.* **2021,** *31*, 2006484.
24. Yu, L.; Zhu, Q.; Song, S.; McElhenny, B.; Wang, D.; Wu, C.; Qin, Z.; Bao, J.; Yu, Y.; Chen, S.; Ren, Z., Non-noble metal-nitride based electrocatalysts for high-performance alkaline seawater electrolysis. *Nat. Commun.* **2019,** *10*, 5106.
25. Wu, L.; Yu, L.; Zhu, Q.; McElhenny, B.; Zhang, F.; Wu, C.; Xing, X.; Bao, J.; Chen, S.; Ren, Z., Boron-modified cobalt iron layered double hydroxides for high efficiency seawater oxidation. *Nano Energy* **2021,** *83*, 105838.
26. Wang, D.; Li, Q.; Han, C.; Lu, Q.; Xing, Z.; Yang, X., Atomic and electronic modulation of self-supported nickel-vanadium layered double hydroxide to accelerate water splitting kinetics. *Nat. Commun.* **2019,** *10*, 3899.
27. Chou, T.-C.; Chang, C.-C.; Yu, H.-L.; Yu, W.-Y.; Dong, C.-L.; Velasco-Vélez, J.-J.; Chuang, C.-H.; Chen, L.-C.; Lee, J.-F.; Chen, J.-M.; Wu, H.-L., Controlling the Oxidation State of the Cu Electrode and Reaction Intermediates for Electrochemical CO_2 Reduction to Ethylene. *J. Am. Chem. Soc.* **2020,** *142*, 2857-2867.
28. Kou, T.; Wang, S.; Hauser, J. L.; Chen, M.; Oliver, S. R. J.; Ye, Y.; Guo, J.; Li, Y., Ni Foam-Supported Fe-Doped β-Ni(OH)2 Nanosheets Show Ultralow Overpotential for Oxygen Evolution Reaction. *ACS Energy Lett.* **2019,** *4*, 622-628.
29. Zhang, H.; Li, X.; Hähnel, A.; Naumann, V.; Lin, C.; Azimi, S.; Schweizer, S. L.; Maijenburg, A. W.; Wehrspohn, R. B., Bifunctional Heterostructure Assembly of NiFe LDH Nanosheets on NiCoP Nanowires for Highly Efficient and Stable Overall Water Splitting. *Adv. Funct. Mater.* **2018,** *28*, 1706847.
30. Chen, G.; Wang, T.; Zhang, J.; Liu, P.; Sun, H.; Zhuang, X.; Chen, M.; Feng, X., Accelerated Hydrogen Evolution Kinetics on NiFe-Layered Double Hydroxide Electrocatalysts by Tailoring Water Dissociation Active Sites. *Adv. Mater.* **2018,** *30*, 1706279.
31. Xu, H.; Fei, B.; Cai, G.; Ha, Y.; Liu, J.; Jia, H.; Zhang, J.; Liu, M.; Wu, R., Boronization-Induced Ultrathin 2D Nanosheets with Abundant Crystalline–Amorphous Phase Boundary

Supported on Nickel Foam toward Efficient Water Splitting. *Adv. Energy Mater.* **2020,** *10*, 1902714.
32. Li, G.; Yang, Q.; Rao, J.; Fu, C.; Liou, S.-C.; Auffermann, G.; Sun, Y.; Felser, C., In Situ Induction of Strain in Iron Phosphide (FeP2) Catalyst for Enhanced Hydroxide Adsorption and Water Oxidation. *Adv. Funct. Mater.* **2020,** *30*, 1907791.
33. Yu, M.; Zhou, S.; Wang, Z.; Zhao, J.; Qiu, J., Boosting electrocatalytic oxygen evolution by synergistically coupling layered double hydroxide with MXene. *Nano Energy* **2018,** *44*, 181-190.
34. Li, J.; Li, L.; Ma, X.; Han, X.; Xing, C.; Qi, X.; He, R.; Arbiol, J.; Pan, H.; Zhao, J.; Deng, J.; Zhang, Y.; Yang, Y.; Cabot, A., Selective Ethylene Glycol Oxidation to Formate on Nickel Selenide with Simultaneous Evolution of Hydrogen. *Adv. Sci.* **2023,** *10*, 2300841.
35. Aladeemy, S. A.; AlRijraji, T. R.; Amer, M. S.; Arunachalam, P.; Al-Mayouf, A. M. J. C. S.; Technology, Electrooxidation of ethylene glycol coupled with hydrogen production on porous NiO/Ni@ NF nanosheet electrocatalysts. **2025,** *15*, 2571-2583.
36. Qi, J.; An, Z.; Li, C.; Chen, X.; Li, W.; Liang, C., Electrocatalytic selective oxidation of ethylene glycol: A concise review of catalyst development and reaction mechanism with comparison to thermocatalytic oxidation process. *Current Opinion in Electrochemistry* **2022,** *32*, 100929.
37. Liu, K.; Wang, Y.; Liu, F.; Liu, C.; Shi, R.; Chen, Y., Selective electrocatalytic reforming of PET-derived ethylene glycol to formate with a Faraday efficiency of 93.2% at industrial-level current densities. *Chemical Engineering Journal* **2023,** *473*, 145292.
38. Fang, Y.; Cai, C.; Yamashita, H.; Qian, X.; Zhao, Y., Efficient and cost-effective electrocatalysts for coproduction of formate through electrocatalytic oxidation of PET-derived ethylene glycol coupled with CO2 reduction. *Catalysis Today* **2026,** *462*, 115544.
39. Zhang, X.; Li, X.; Zhang, X.; Lu, H.; Sun, Y.; Duan, X.; Ren, Y., Hierarchical CoFe-LDH with nanoneedle-assembled architecture for selective and efficient electrocatalytic oxidation of ethylene glycol to formate. *Journal of Electroanalytical Chemistry* **2025,** *998*, 119532.
40. Lv, W.; Kong, X.; Shi, H.; Pan, L.; Liu, R.; Wang, C.; Wei, M.; Zhang, R.; Wang, W., 3D NiCo bimetallic oxide nanowires for ethylene glycol electrooxidation with formate reoxidation suppression via concentration gradient strategy. *Inorganic Chemistry Communications* **2025,** *182*, 115605.
41. Ma, Y.; Ge, H.; Zhang, Y.; Jian, N.; Yu, J.; Arbiol, J.; Li, C.; Zhong, Y.; Li, L.; Kang, H.; Wang, J.; Cabot, A.; Li, J., Selective Electrooxidation of Ethylene Glycol to Formate with Hydrogen Cogeneration in Ni3S2 Nanodomains on NiFeMn-LDH Nanosheet Arrays. *ACS Sustainable Chemistry & Engineering* **2025,** *13*, 5601-5612.
42. Luo, H.; Xie, X.; Sun, J.; Guo, S.; Khalakhan, I. J. J. o. M. C. A., Effective and selective ethylene glycol electrooxidation with compositionally controlled Pt–Au bimetallic electrocatalysts. **2026,** *14*, 343-347.
43. Wang, Y.; Liu, K.; Liu, F.; Liu, C.; Shi, R.; Chen, Y. J. G. C., Selective electro-reforming of waste polyethylene terephthalate-derived ethylene glycol into C 2 chemicals with long-term stability. **2023,** *25*, 5872-5877.
44. Ma, H.-Z.; He, S.-H.; Zhang, Y.; Wang, L.; Yi, Y.-N.; Yang, Y.-Y., Selective Electrocatalytic Oxidation of Ethylene Glycol into Glycolic Acid at Coin-Group Electrodes: An Investigation on Catalytic Activity and Selectivity. *ACS Sustainable Chemistry & Engineering* **2024,** *12*, 12249-12259.
45. Li, Y.; Liao, Q.; Ji, P.; Jie, S.; Wu, C.; Tong, K.; Zhu, M.; Zhang, C.; Li, H., Accelerated Selective Electrooxidation of Ethylene Glycol and Inhibition of C–C Dissociation Facilitated by Surficial Oxidation on Hollowed PtAg Nanostructures via In Situ Dynamic Evolution. *JACS Au* **2025,** *5*, 714-726.

46. Liu, Y.; Wang, L.; Zhang, Y.; Xie, J.; Li, J.; Wei, J.; Zhang, M.; Yang, Y., From Ethylene Glycol to Glycolic Acid: Electrocatalytic Conversion on Pt-Group Metal Surfaces. *Inorg. Chem.* **2024,** *63*, 14794-14803.
47. Watson, N. I.; Fehler, A.; Stoop, M.; van den Bosch, B.; Rothenberg, G., Elemental Trade-Off in the Selective Electro-Oxidation of Ethylene Glycol on Palladium-Silver/Nickel Electrodes. *ChemSusChem* **2025,** *18*, e202500724.
48. Xia, S.; Wu, F.; Liu, Q.; Gao, W.; Guo, C.; Wei, H.; Hussain, A.; Zhang, Y.; Xu, G.; Niu, W., Steering the Selective Production of Glycolic Acid by Electrocatalytic Oxidation of Ethylene Glycol with Nanoengineered PdBi-Based Heterodimers. *Small* **2024,** *20*, 2400939.
49. Shi, R.; Wang, Y.; Chen, J.; Liu, F.; Sun, T.; Tse, E. C. M.; Chen, Y., Electrooxidation of Ethylene Glycol to Glycolic Acid in a Neutral Electrolyte via Enhanced *OH Generation and Directional Spillover. *J. Am. Chem. Soc.* **2026**.
50. Li, Z.; Wang, S.; Yin, Y.; Qin, R.; Wei, C.; Luo, H.; Mu, T., Electrooxidation of Ethylene Glycol to Glycolic Acid with Pt-Ni(OH)2 Catalysts: High Efficiency and Selectivity for PET Plastics Upgrading. *Chemistry – An Asian Journal* **2025,** *20*, e202401843.
51. Shi, K.; Si, D.; Teng, X.; Chen, L.; Shi, J., Pd/NiMoO4/NF electrocatalysts for the efficient and ultra-stable synthesis and electrolyte-assisted extraction of glycolate. *Nat. Commun.* **2024,** *15*, 2899.
